# Supplementary material for: Sintering-induced cation displacement in protonic ceramics and way for its suppression
Source: Nat Commun. 2023 Dec 2;14:7984. doi: 10.1038/s41467-023-43725-x (PMC10693594; doi:10.1038/s41467-023-43725-x)
Supplement: Supplementary file 1 — Supplementary Information [file 41467_2023_43725_MOESM1_ESM.pdf]

Supplementary Information

Sintering-induced cation displacement in protonic ceramics and way for its suppression

Ze Liu[1,2], Yufei Song[3], Xiaolu Xiong[1], Yuxuan Zhang[1], Jingzeng Cui[1,2], Jianqiu Zhu[1,2], Lili Li[4], Jing Zhou[1], Chuan Zhou[5], Zhiwei Hu[6], Guntae Kim[1], Francesco Ciucci[7], Zongping Shao[8]*, Jian-Qiang Wang[1,2]*, Linjuan Zhang[1,2]*

[1] Key Laboratory of Interfacial Physics and Technology, Shanghai Institute of Applied Physics, Chinese Academy of Sciences, Shanghai 201800, China.

[2] University of Chinese Academy of Sciences, Beijing 100049, China.

[3] Department of Mechanical and Aerospace Engineering, The Hong Kong University of Science and Technology, Clear Water Bay, Hong Kong, China.

[4] State Key Laboratory of Crystal Materials and Institute of Crystal Materials, Shandong University, Jinan, 250100, China.

[5] State Key Laboratory of Materials-Oriented Chemical Engineering, College of Chemical Engineering, Nanjing Tech University, Nanjing, 211816, China.

[6] Max Planck Institute for Chemical Physics of Solids, Dresden 01187, Germany.

[7] Chair of Electrode Design for Electrochemical Energy Storage Systems, University of Bayreuth, 95448, Bayreuth, 9 Germany.

[8] WA School of Mines: Minerals, Energy and Chemical Engineering, Curtin University, Perth, WA6845, Australia.

Corresponding author

*Linjuan Zhang: email: zhanglinjuan@sinap.ac.cn;

*Jian-Qiang Wang: email: wangjianqiang@sinap.ac.cn;

*Zongping Shao: email: zongping.shao@curtin.edu.au

Supplementary Figures

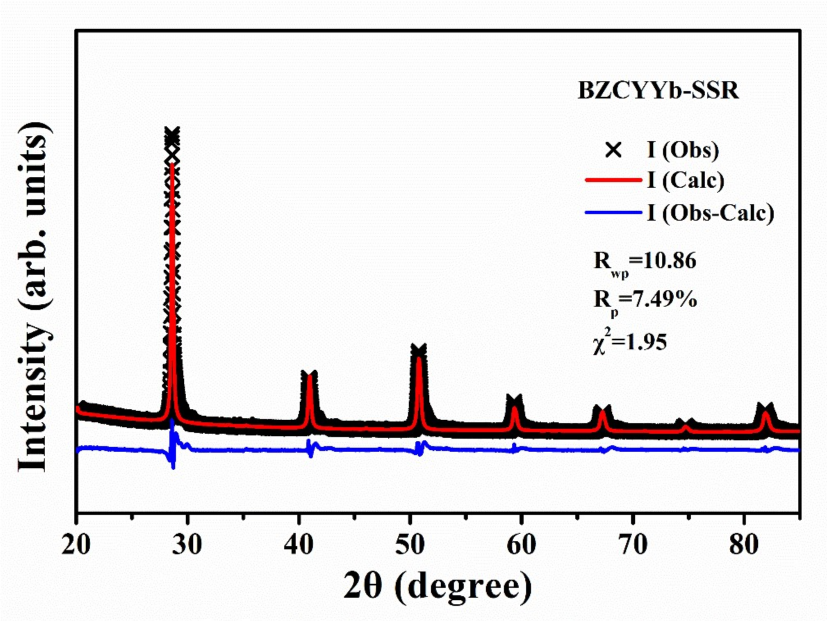

Supplementary Figure 1. Rietveld refinement plots of the XRD pattern of BZCYYb-SSR synthesized at 1000 °C.

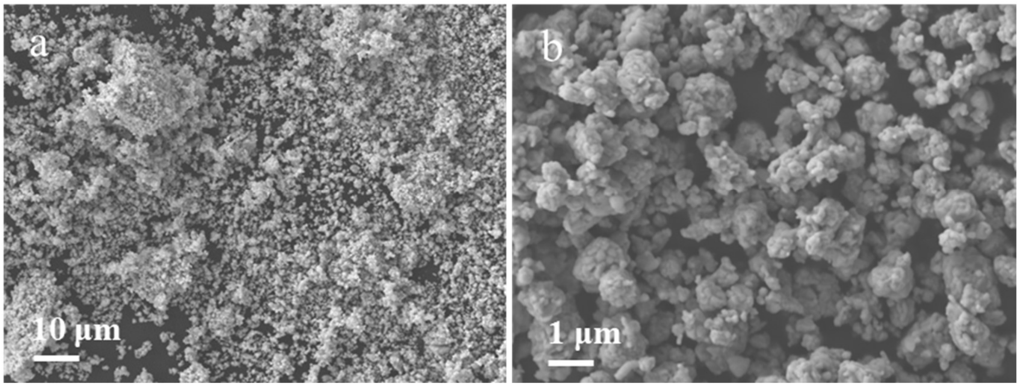

Supplementary Figure 2. a, b The SEM images of BZCYYb-SSR powder.

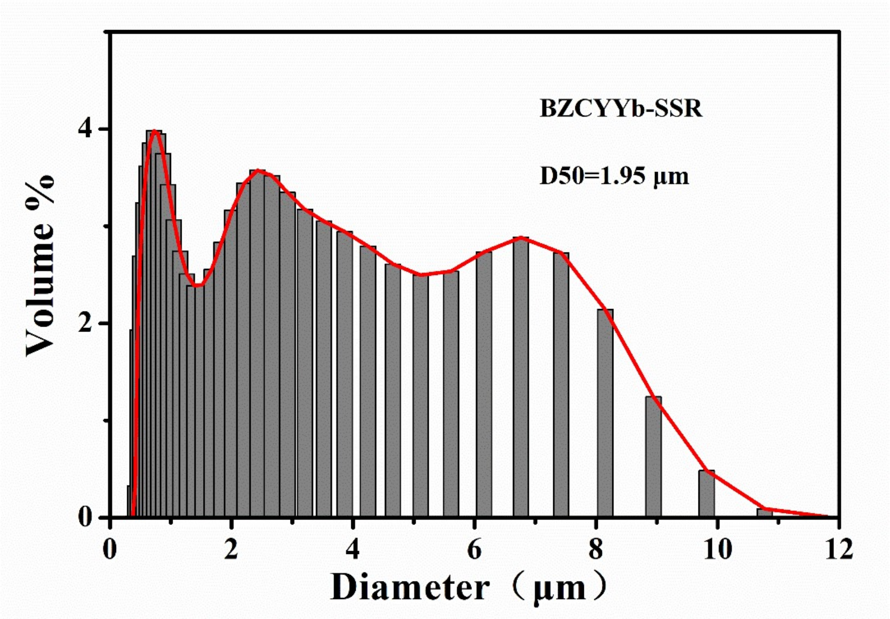

Supplementary Figure 3. Particle size analysis of BZCYYb-SSR.

Supplementary Discussion: To investigate the particle size distribution, the BZCYYb-SSR sample was investigated by a laser particle size analyzer. And the key parameter D50 of the BZCYYb-SSR sample was 1.95 µm. D50 is a typical index for evaluating the particle size of powder and is usually used to represent the average particle size of the sample. The particle size distribution of the BZCYYb-SSR sample was asymmetrical with a large particle size distribution range (0-12 µm), indicating that the particle size of BZCYYb powder prepared by traditional solid state reaction method was not uniform.

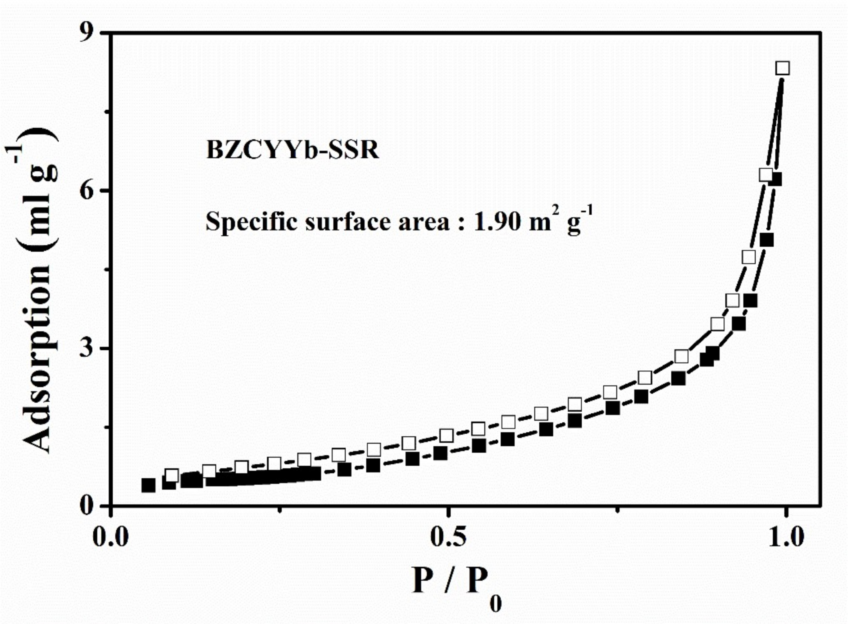

Supplementary Figure 4. The N_2 adsorption-desorption isotherm of pristine BZCYYb-SSR powder.

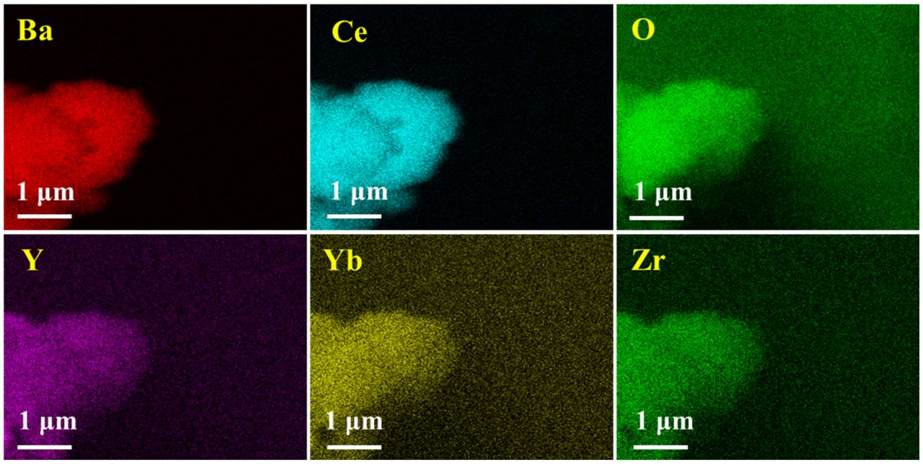

Supplementary Figure 5. The EDS mapping of Ba, Ce, O, Y, Yb, Zr of BZCYYb-SSR powder.

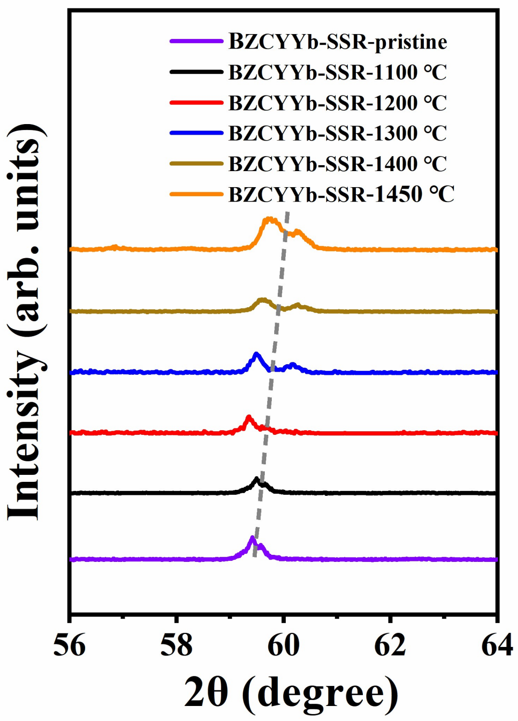

Supplementary Figure 6. Magnified XRD patterns of BZCYYb-SSR pristine sample and after quenching at different temperatures.

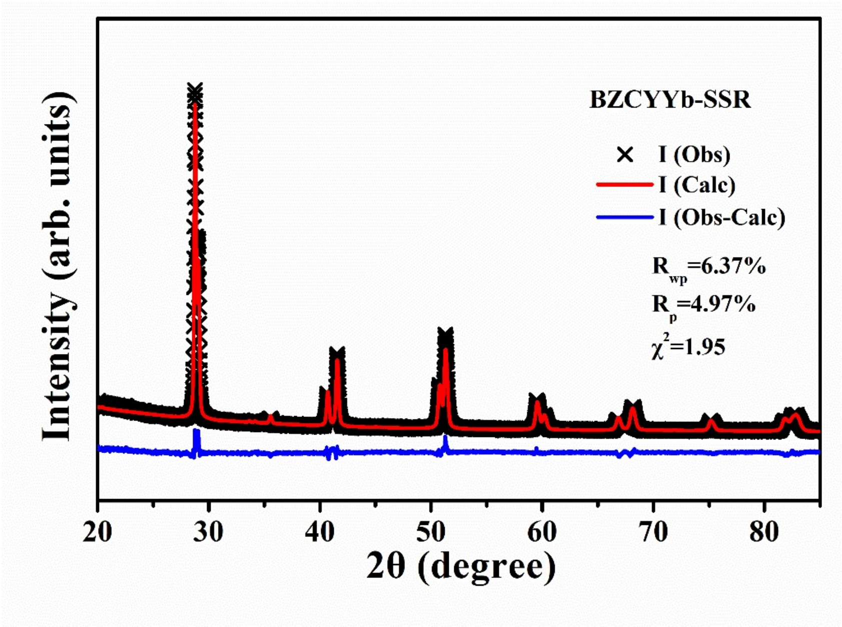

Supplementary Figure 7. Rietveld refinement plots of the XRD patterns of BZCYYb-SSR quenched at 1300 °C.

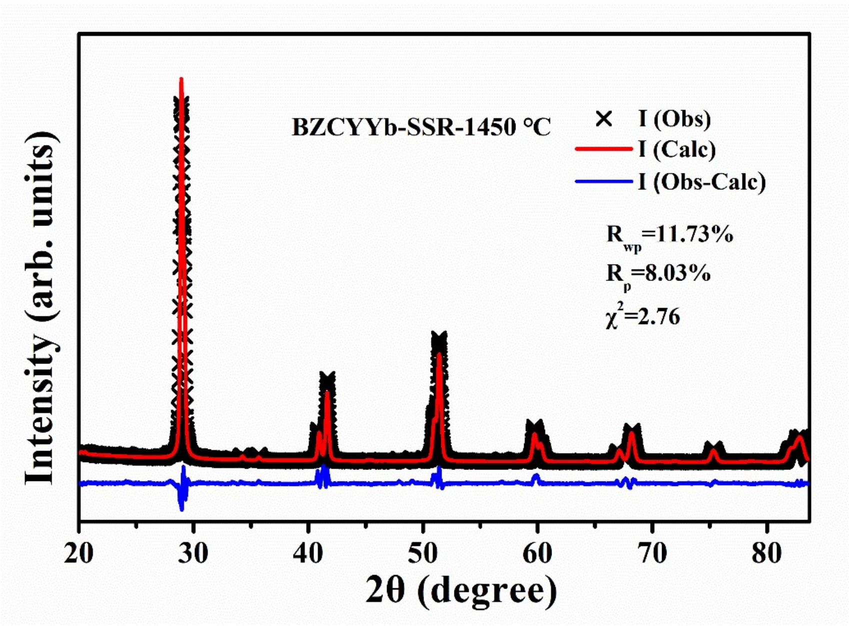

Supplementary Figure 8. Rietveld refinement plots of the XRD patterns of BZCYYb-SSR quenched at 1450 ℃.

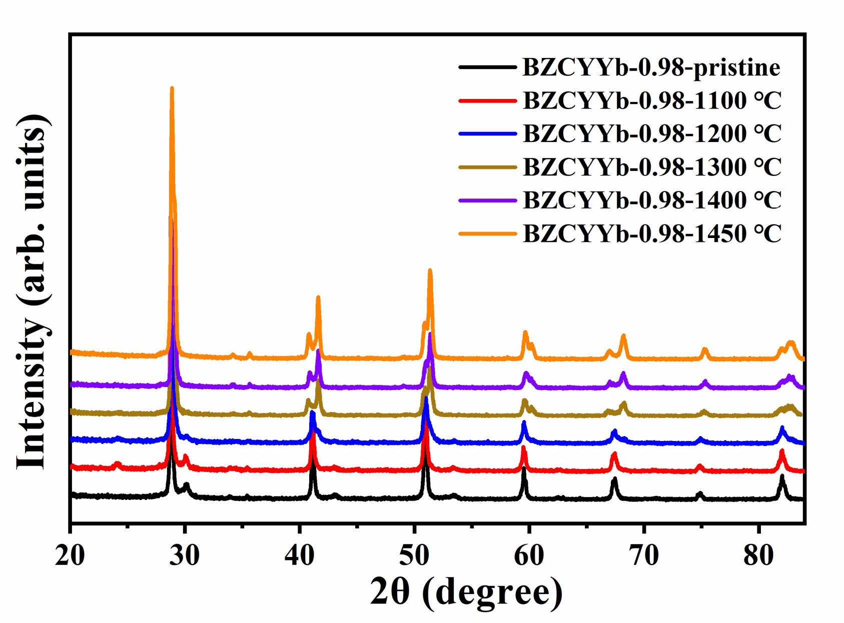

Supplementary Figure 9. XRD patterns of pristine BZCYYb-0.98-SSR and quenched at different temperatures.

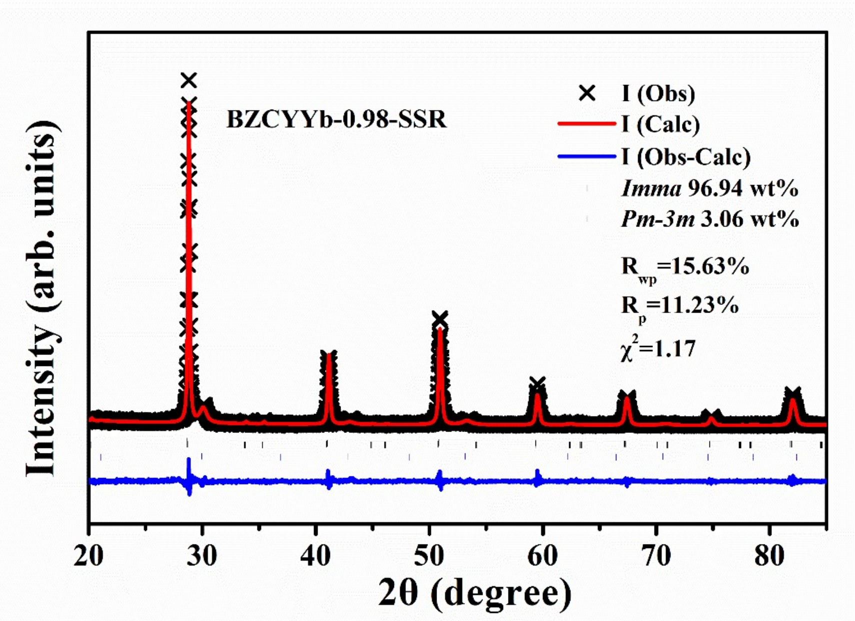

Supplementary Figure 10. Rietveld refinement plots of the XRD pattern of BZCYYb-0.98-SSR calcined at 1000 ℃. The as-synthesized BZCYYb-0.98-SSR consists of two different crystalline phases, the main phase is BZCYYb-0.98 (space group: *Imma*), and the other phase is $BaZrO_3$ (space group: *Pm-3m*).

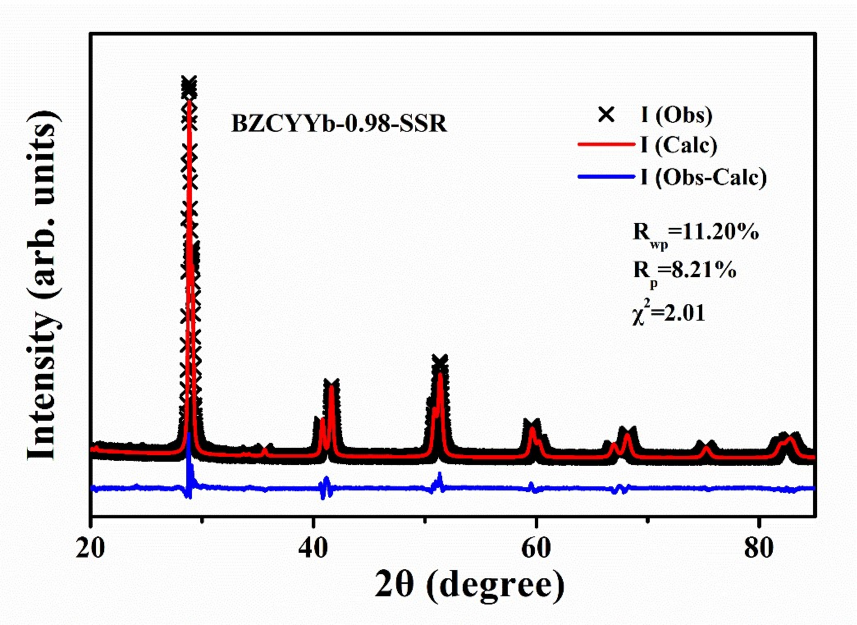

Supplementary Figure 11. Rietveld refinement plots of the XRD pattern of BZCYYb-0.98-SSR quenched at 1300 °C.

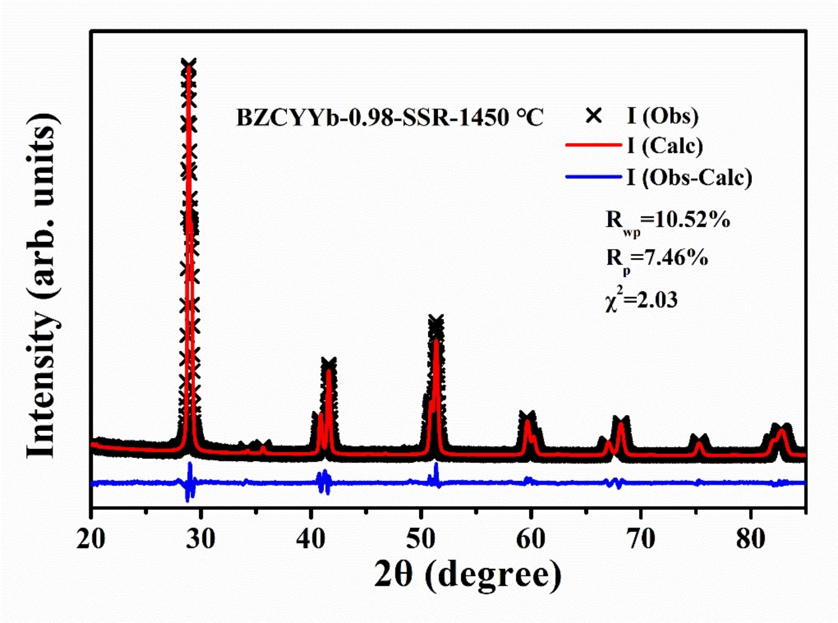

Supplementary Figure 12. Rietveld refinement plots of the XRD patterns of BZCYYb-0.98-SSR quenched at 1450 ℃.

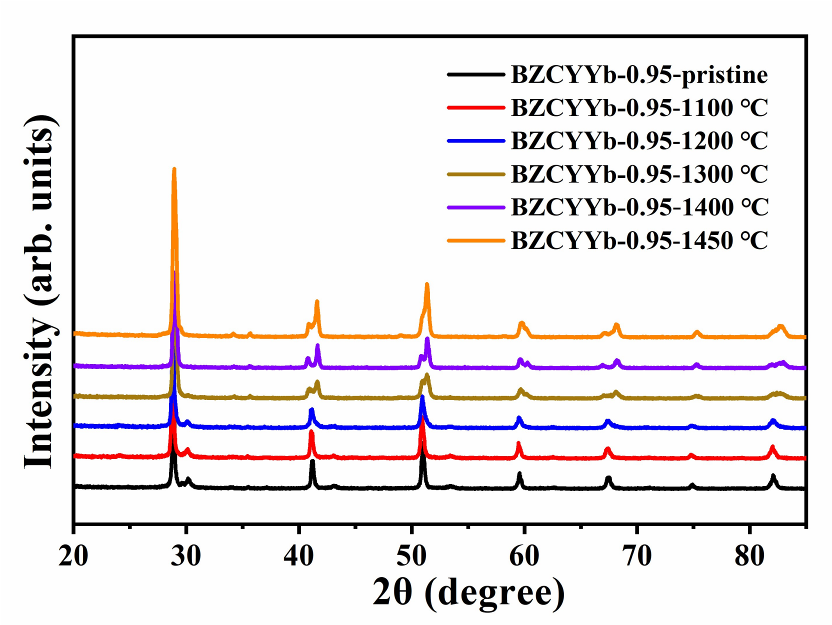

Supplementary Figure 13. XRD patterns of pristine BZCYYb-0.95-SSR and quenched at different temperatures.

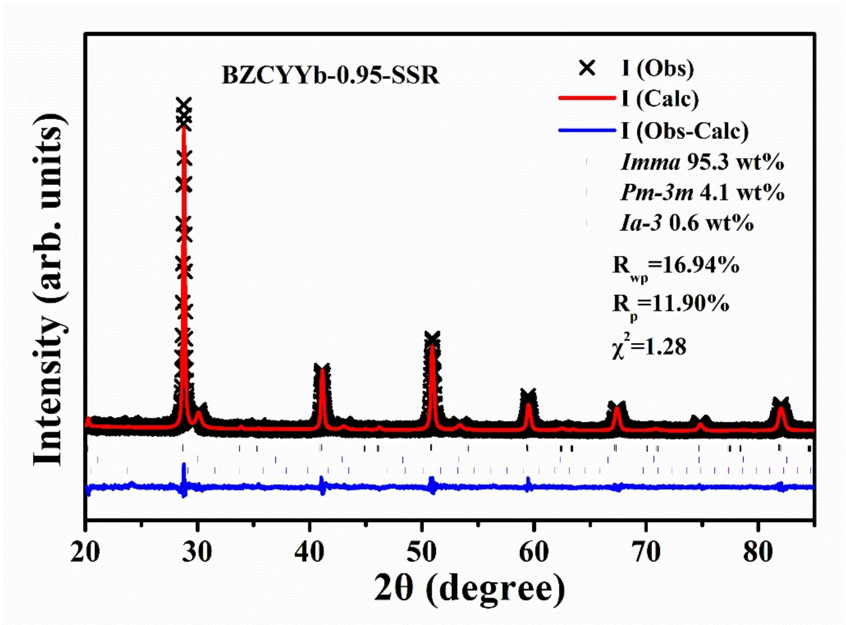

Supplementary Figure 14. Rietveld refinement plots of the XRD pattern of BZCYYb-0.95-SSR calcined at 1000 °C. The as-synthesized BZCYYb-0.95-SSR consists of three different crystalline phases, of which the main phase is BZCYYb-0.95 (space group: *Imma*), the second phase is $BaZrO_3$ (space group: *Pm-3m*), and the third phase is Y_2O_3 (space group: *Ia-3*).

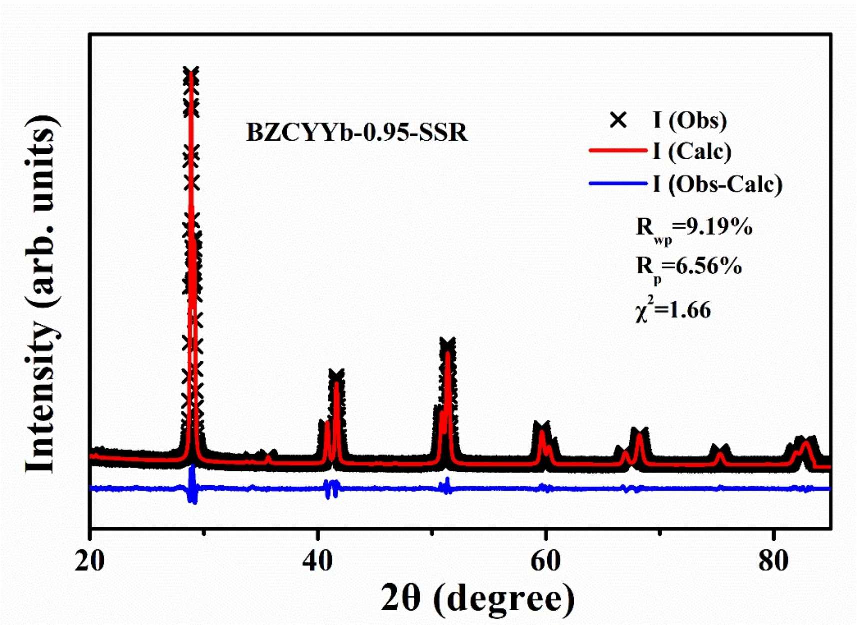

Supplementary Figure 15. Rietveld refinement plots of the XRD pattern of BZCYYb-0.95-SSR quenched at 1300 ℃.

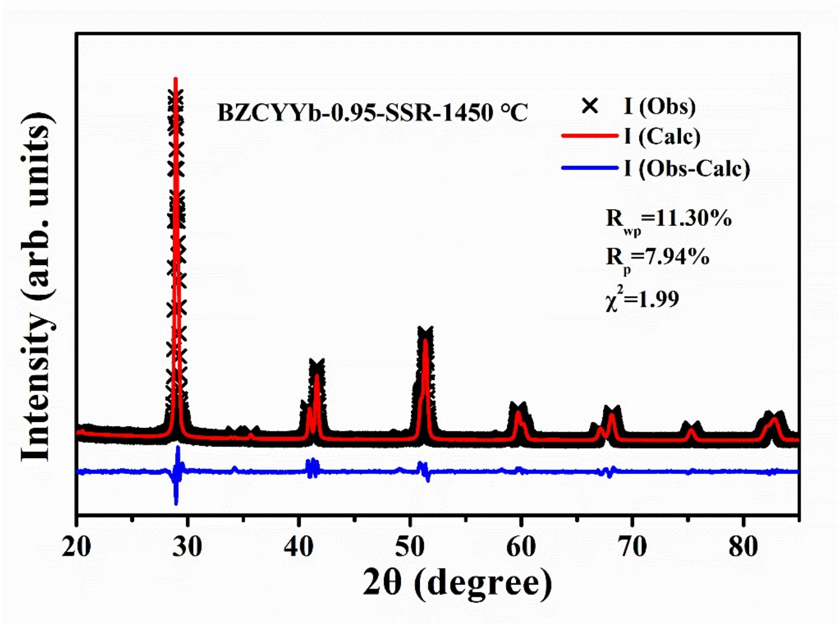

Supplementary Figure 16. Rietveld refinement plots of the XRD patterns of BZCYYb-0.95-SSR quenched at 1450 ℃.

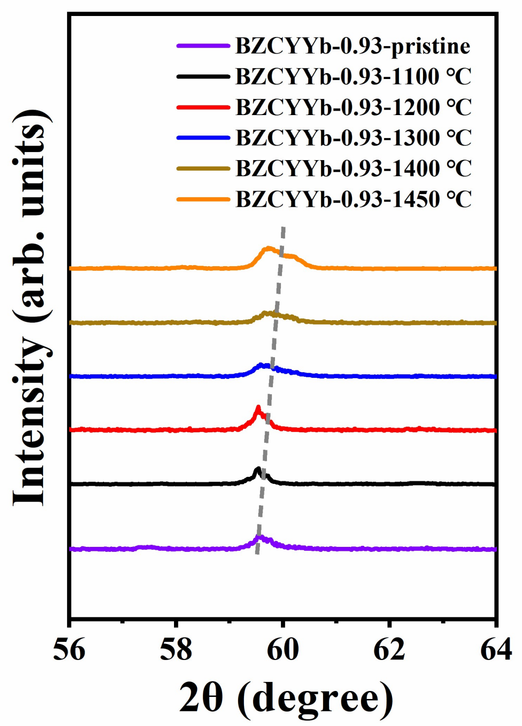

Supplementary Figure 17. Magnified XRD patterns of BZCYYb-0.93-SSR pristine sample and after quenching at different temperatures.

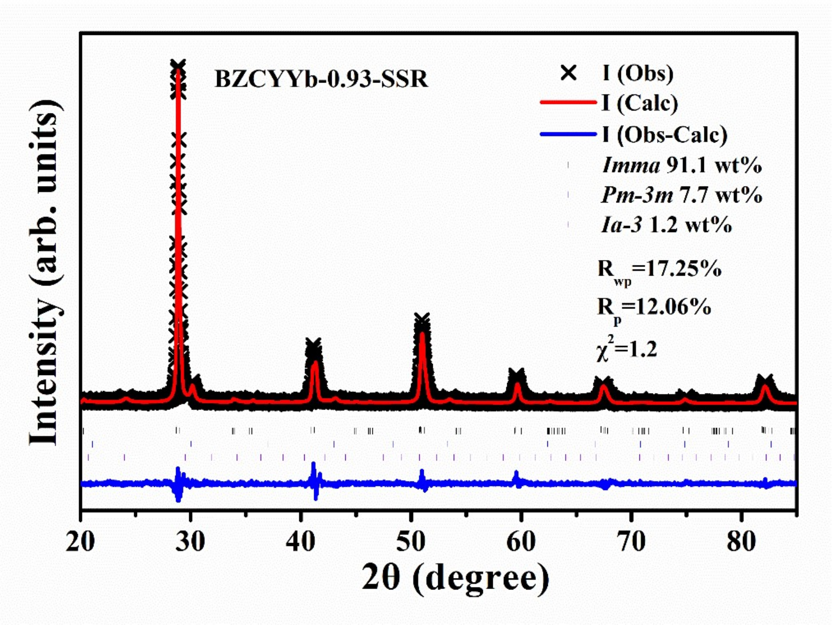

Supplementary Figure 18. Rietveld refinement plots of the XRD pattern of BZCYYb-0.93-SSR calcined at 1000 ℃.

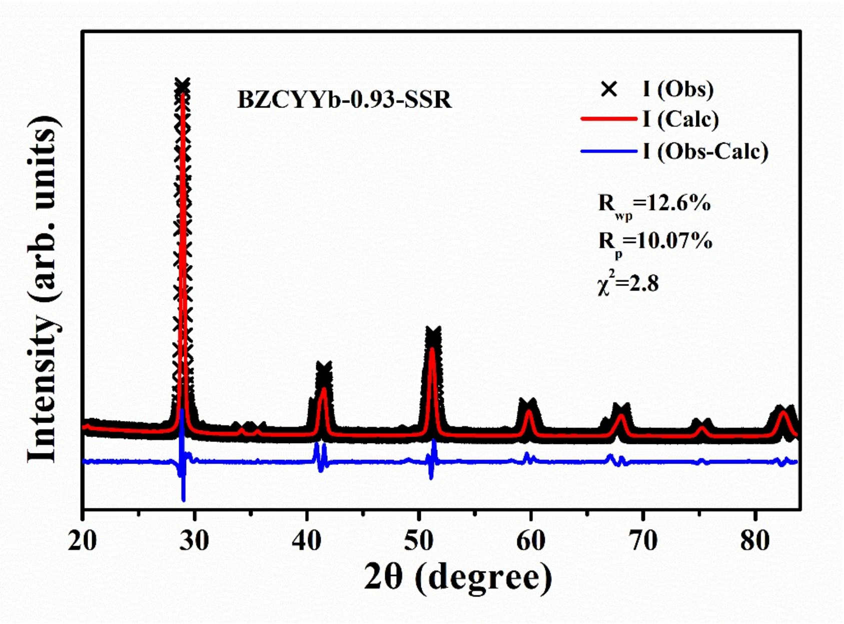

Supplementary Figure 19. Rietveld refinement plots of the XRD pattern of BZCYYb-0.93-SSR quenched at 1300 ℃.

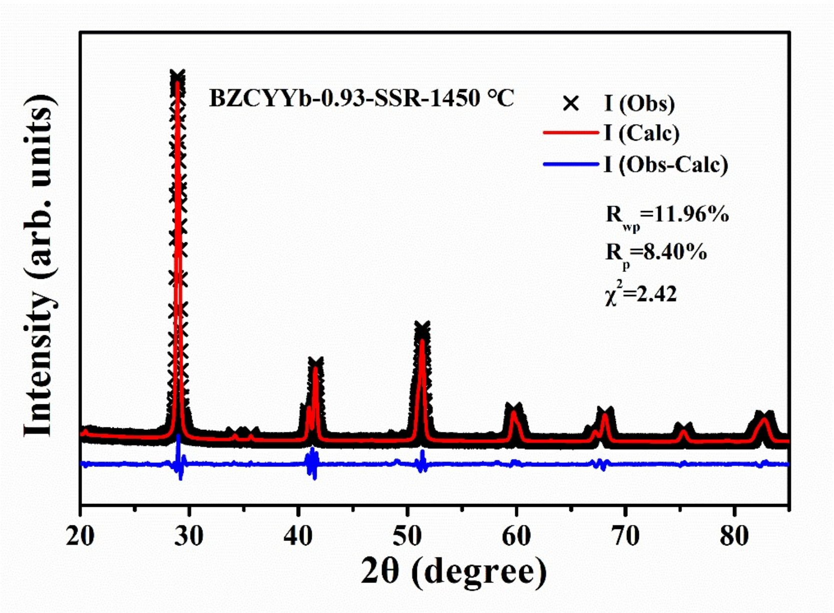

Supplementary Figure 20. Rietveld refinement plots of the XRD pattern of BZCYYb-0.93-SSR quenched at 1450 ℃.

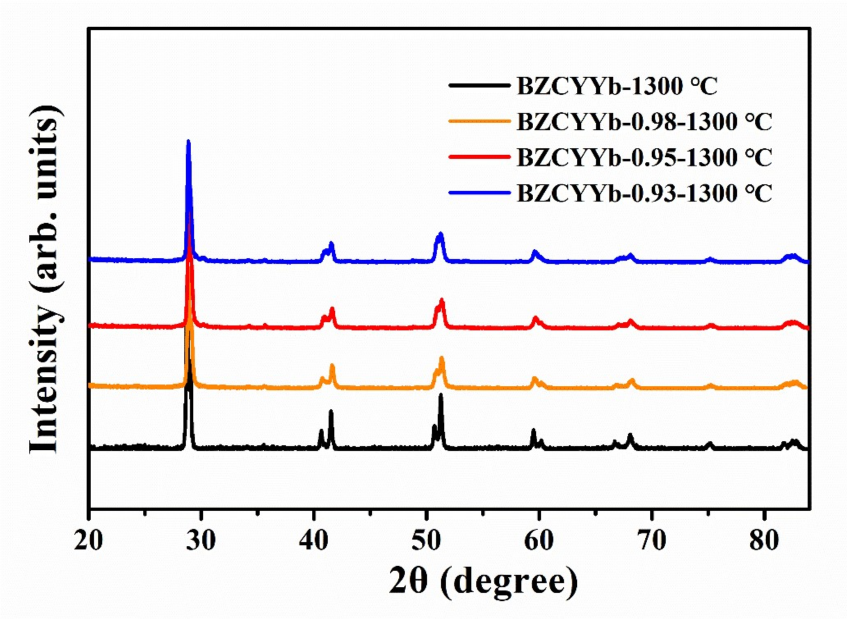

Supplementary Figure 21. XRD patterns of BZCYYb, BZCYYb-0.98, BZCYYb-0.95 and BZCYYb-0.93 prepared by solid state reaction method after quenching at 1300 °C.

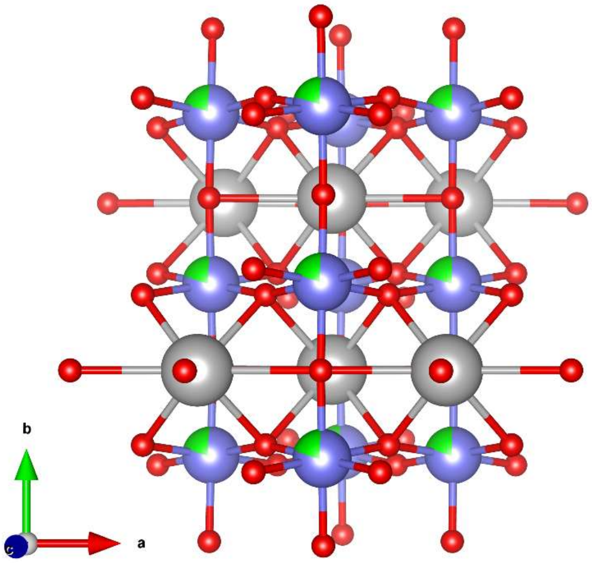

Supplementary Figure 22. Crystal structure of $BaZr_{0.2}Ce_{0.8}O_{3-\delta}$ perovskite with an orthorhombic structure (*Imma*). Grey balls: Ba atoms; Red balls: O atoms; Purple/green balls: Ce/Zr atoms, Zr replaces 1/5 of the Ce atoms.

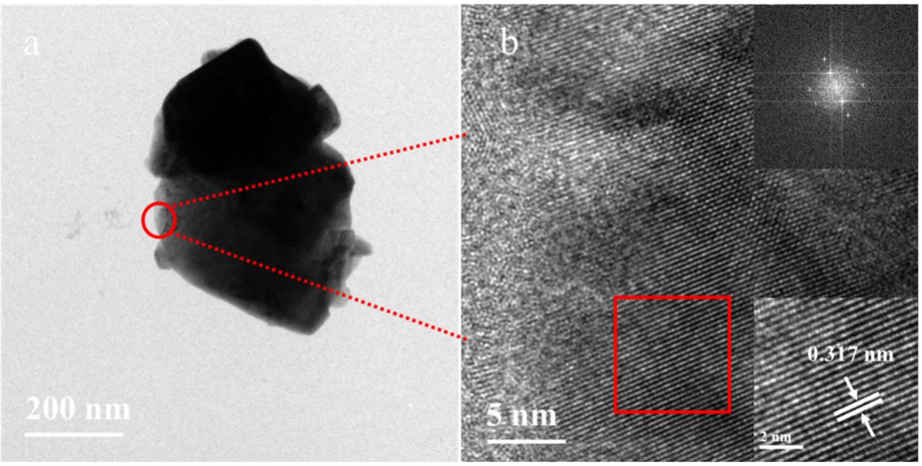

Supplementary Figure 23. a, b TEM images of BZCYYb-SSR powder calcined at 1000 ℃. Inset images are the corresponding fast Fourier transform (FFT) pattern of the area in the red box.

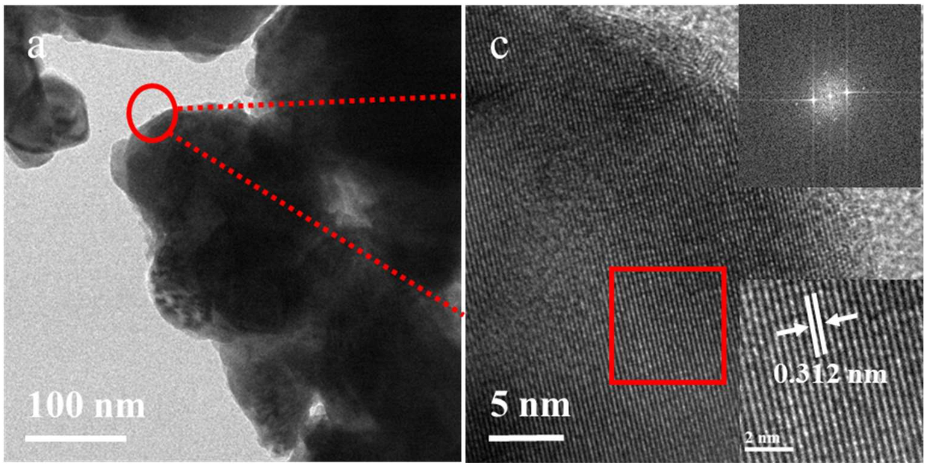

Supplementary Figure 24. a, b TEM images of BZCYYb-SSR-0.93 powder calcined at 1000 ℃. Inset images are the corresponding fast Fourier transform (FFT) pattern of the area in the red box.

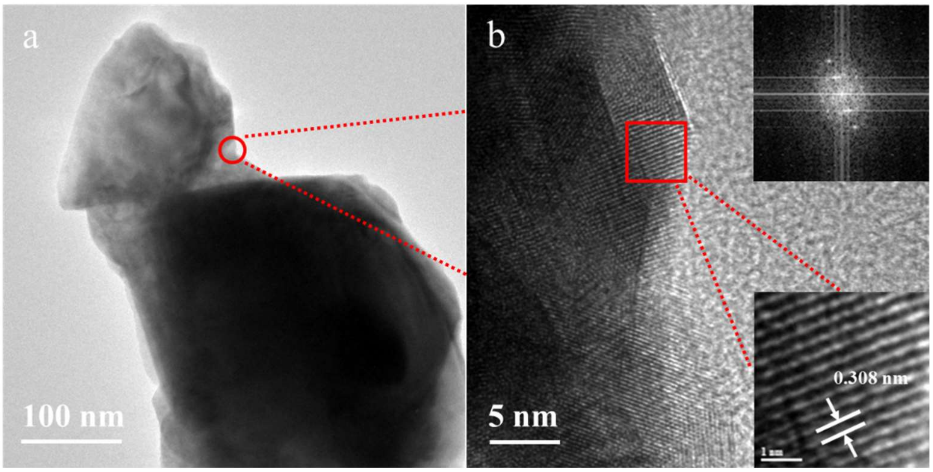

Supplementary Figure 25. a, b TEM images of BZCYYb-SSR powder quenched at 1300 ℃. Inset images are the corresponding fast Fourier transform (FFT) pattern of the area in the red box.

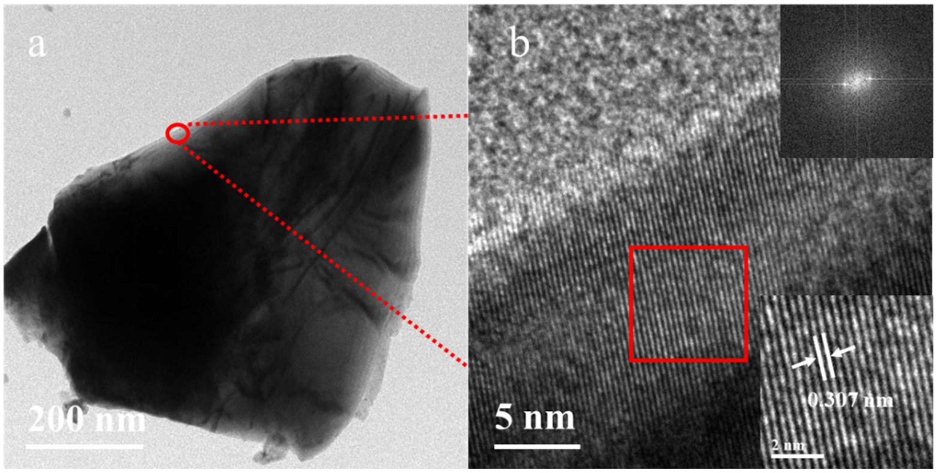

Supplementary Figure 26. a, b TEM images of BZCYYb-SSR powder quenched at 1450 °C. Inset images are the corresponding fast Fourier transform (FFT) pattern of the area in the red box.

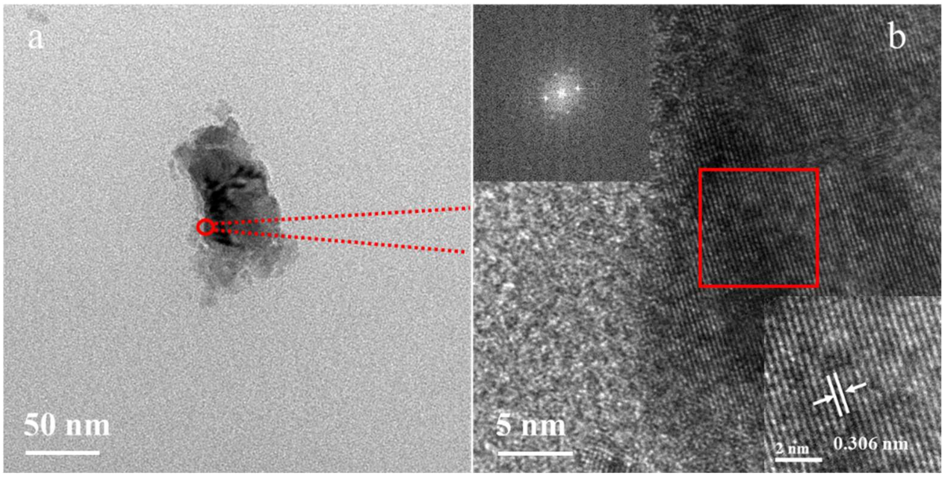

Supplementary Figure 27. a, b TEM images of BZCYYb-SSR-0.93 powder quenched at 1300 ℃. Inset images are the corresponding fast Fourier transform (FFT) pattern of the area in the red box.

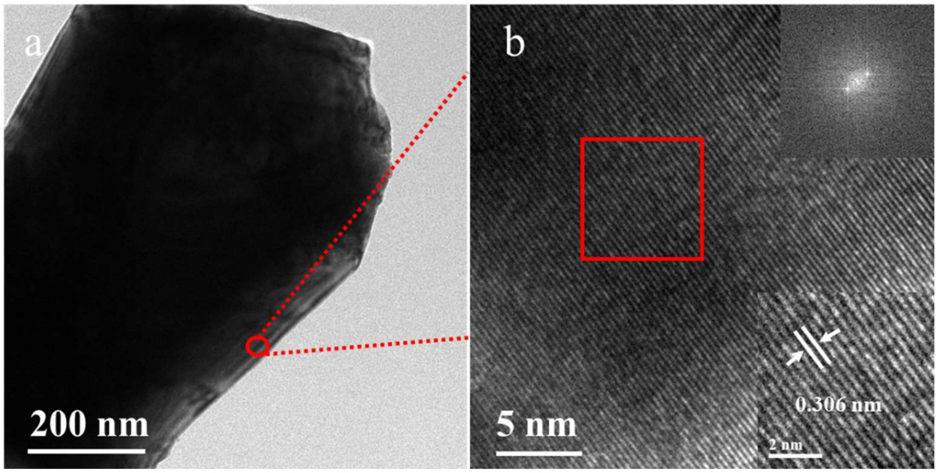

Supplementary Figure 28. a, b TEM images of BZCYYb-SSR-0.93 powder quenched at 1450 ℃. Inset images are the corresponding fast Fourier transform (FFT) pattern of the area in the red box.

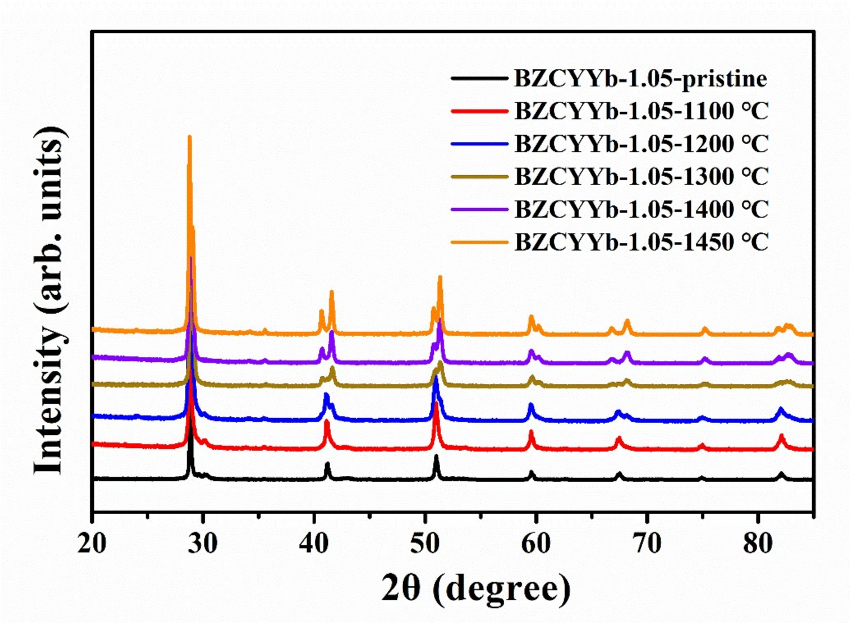

Supplementary Figure 29. XRD patterns of pristine BZCYYb-1.05-SSR sample and after quenching at different temperatures.

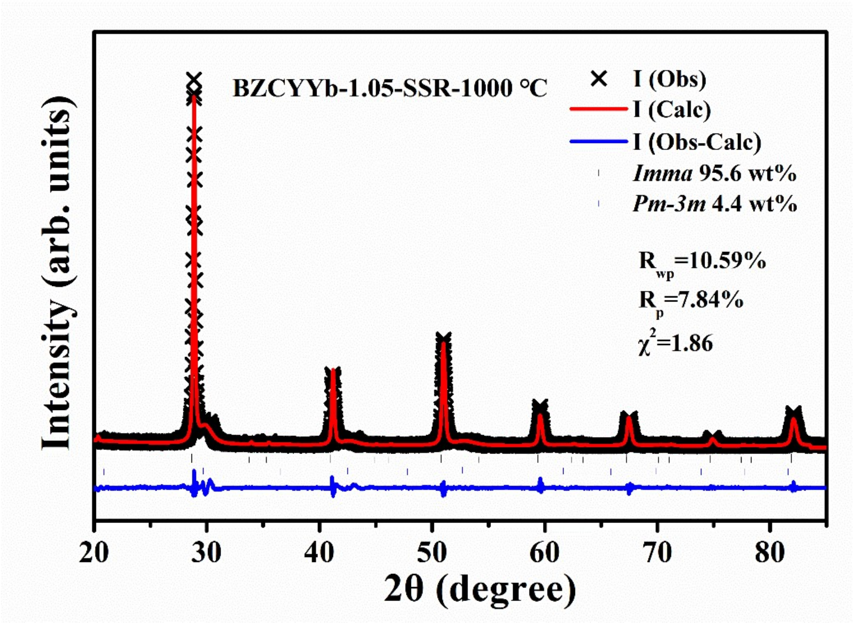

Supplementary Figure 30. Rietveld refinement plots of the XRD pattern of pristine BZCYYb-1.05-SSR synthesized at 1000 °C.

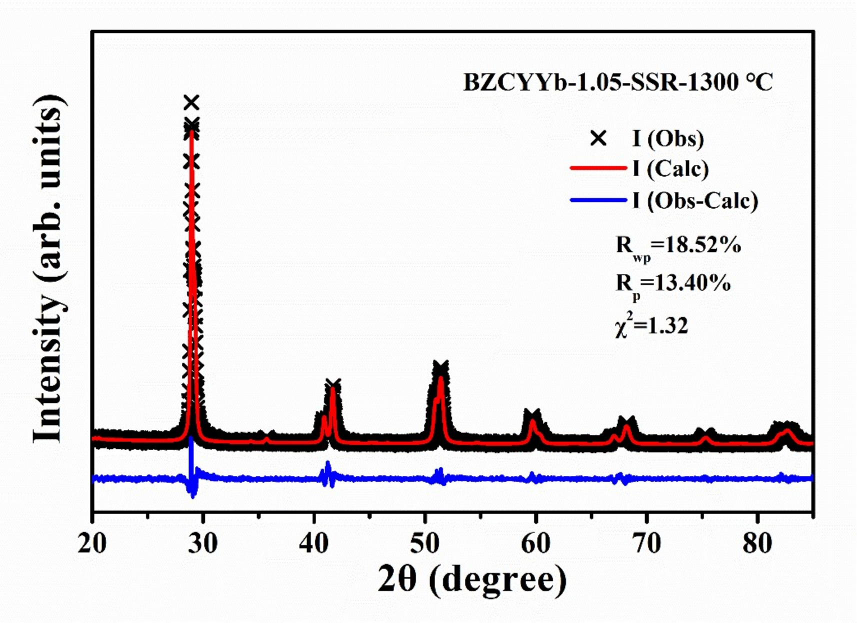

Supplementary Figure 31. Rietveld refinement plots of the XRD pattern of BZCYYb-1.05-SSR quenched at 1300 ℃.

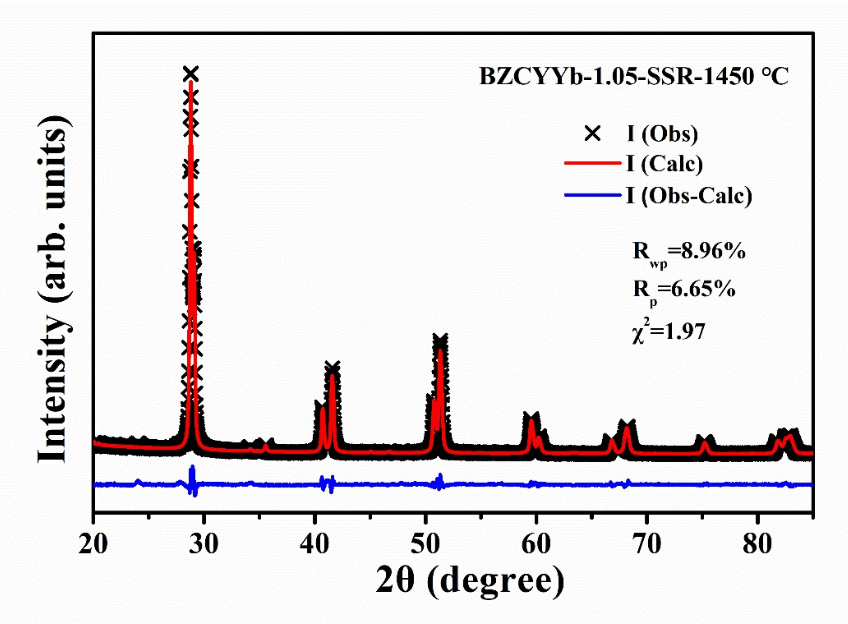

Supplementary Figure 32. Rietveld refinement plots of the XRD pattern of BZCYYb-1.05-SSR quenched at 1450 ℃.

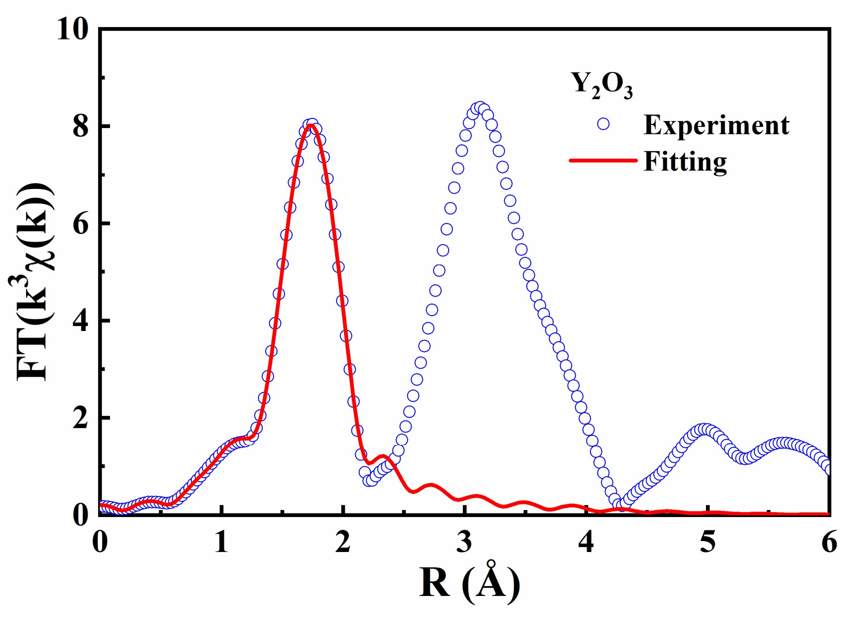

Supplementary Figure 33. Fourier-transformed EXAFS data measured at the Y *K*-edge and its fitting curve for Y_2O_3 reference.

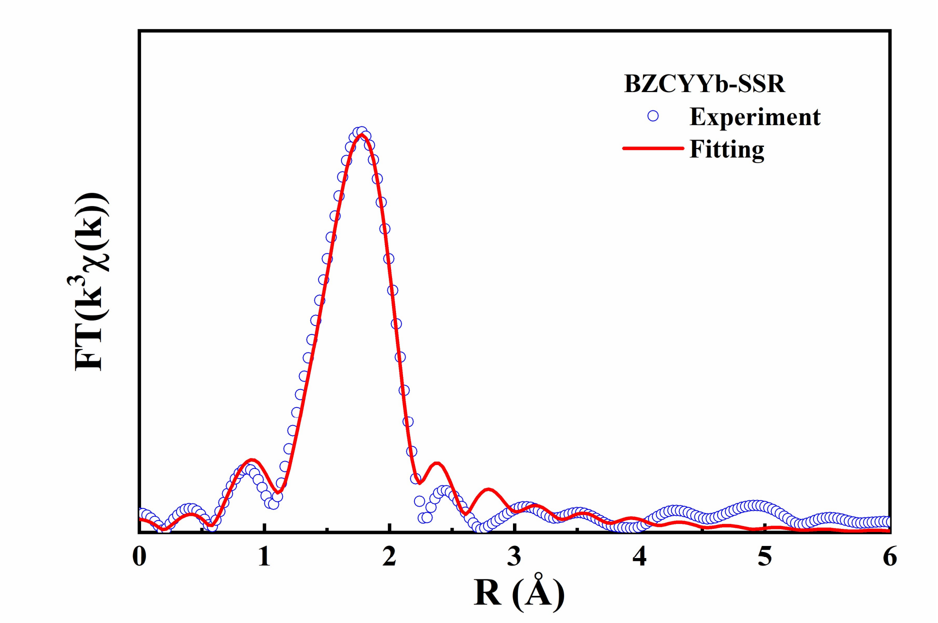

Supplementary Figure 34. Fourier-transformed EXAFS data measured at the Y *K*-edge and its fitting curve for BZCYYb-SSR.

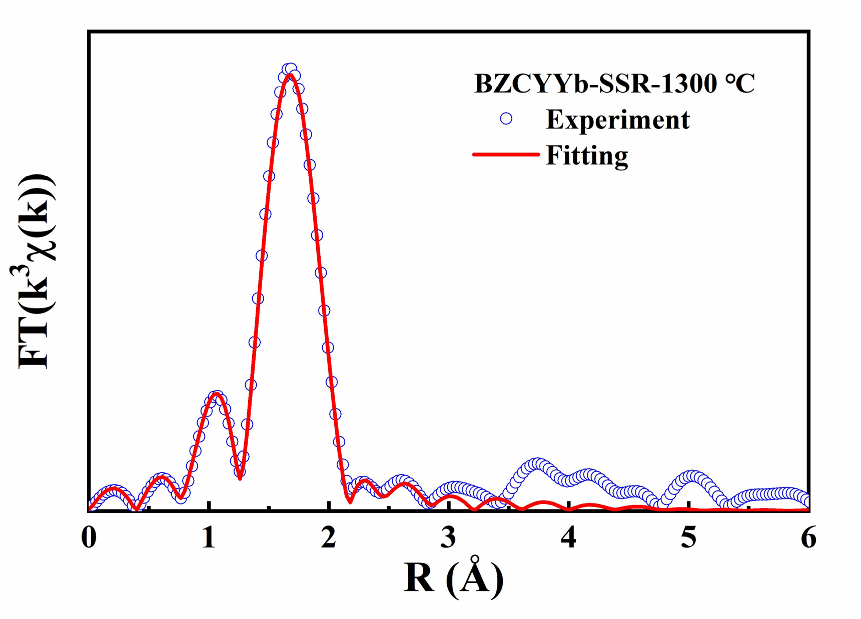

Supplementary Figure 35. Fourier-transformed EXAFS data measured at the Y *K*-edge and its fitting curve for BZCYYb-SSR after quenching at 1300 °C.

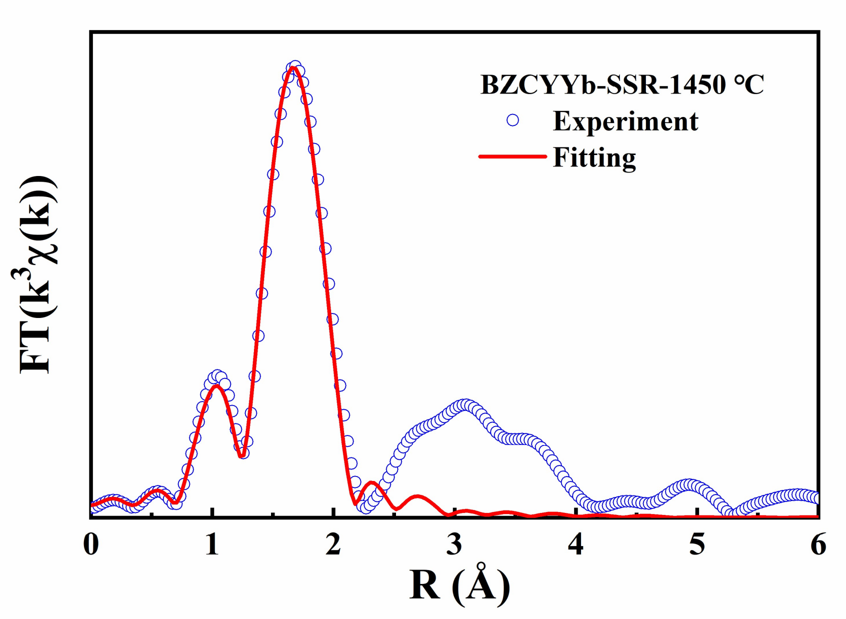

Supplementary Figure 36. Fourier-transformed EXAFS data measured at the Y *K*-edge and its fitting curve for BZCYYb-SSR after quenching at 1450 °C.

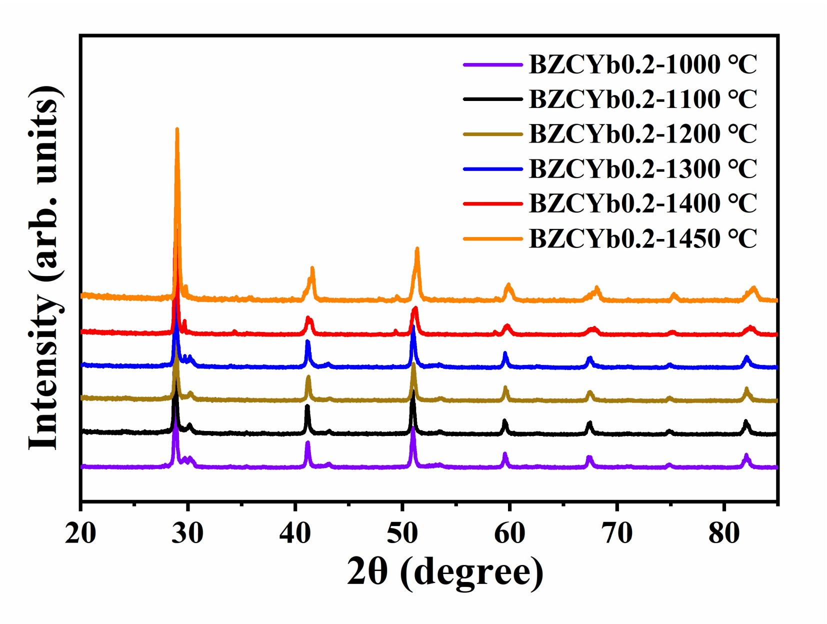

Supplementary Figure 37. XRD patterns of pristine BZCYb0.2-SSR sample and after quenching at different temperatures.

Supplementary Discussion: $BaZr_{0.1}Ce_{0.7}Yb_{0.2}O_{3-\delta}$ sample with Yb content of 20% was prepared by solid state reaction method (BZCYb0.2-SSR), and the treatments at the same temperatures as BZCYYb-SSR were conducted (**Supplementary Figure 37**). However, when the temperature reached 1400 °C, some impurities were detected, and the impurity phase was Yb_2O_3 (**Supplementary Figure 38**). This result indicated that Yb^{3+} preferentially precipitates in the form of secondary phase Yb_2O_3 during high temperature sintering process.

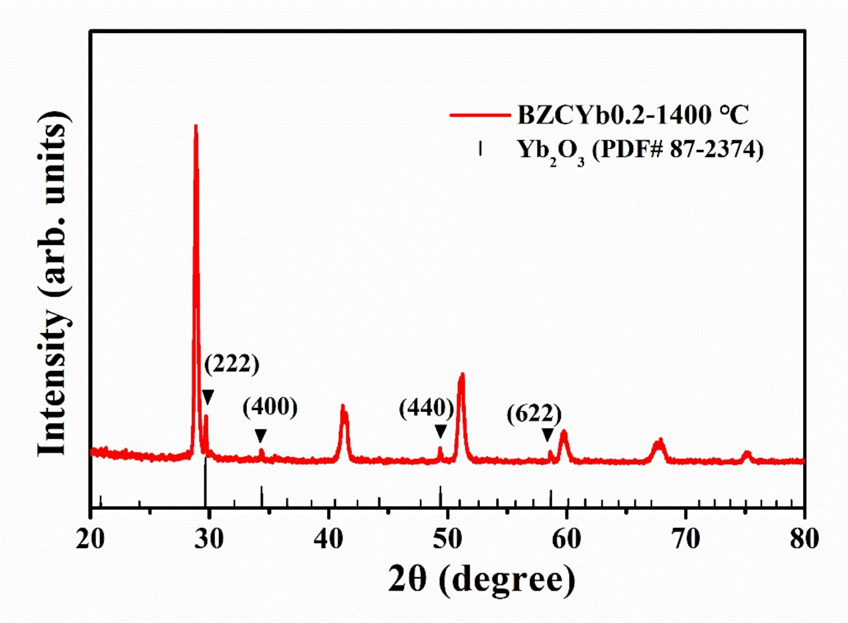

Supplementary Figure 38. XRD pattern of pristine BZCYb0.2-SSR sample after quenching at 1400 °C.

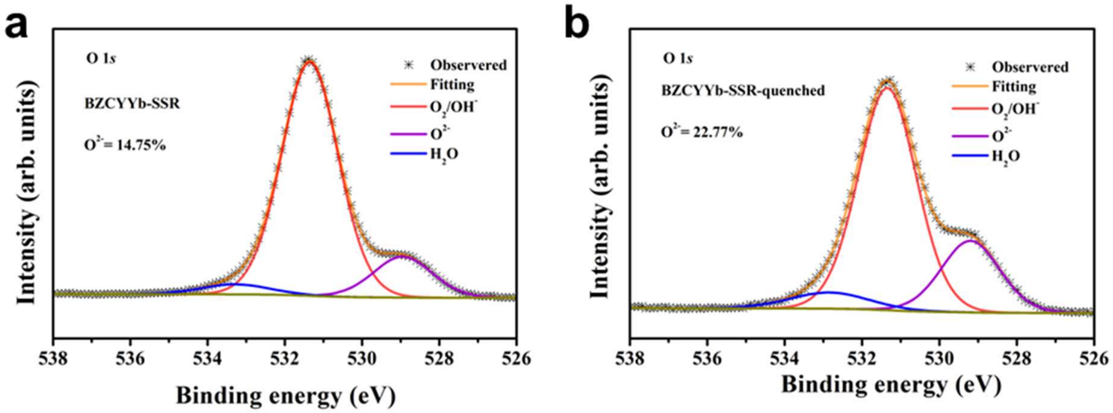

Supplementary Figure 39. a, b The XPS spectra of O 1*s* for BZCYYb-SSR before and after quenching.

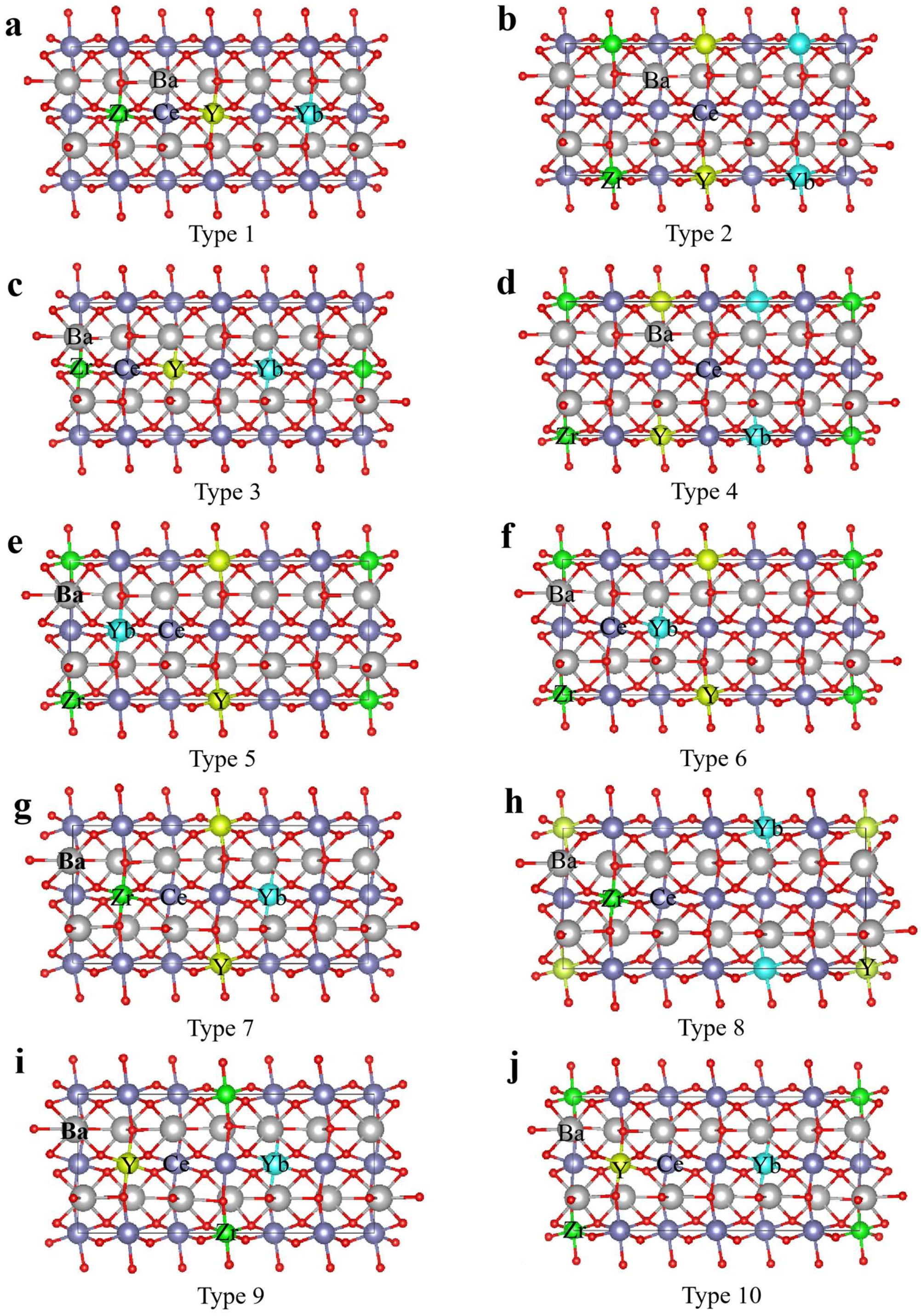

Supplementary Figure 40. Different configurations of $BaZr_{0.1}Ce_{0.7}Y_{0.1}Yb_{0.1}O_3$.

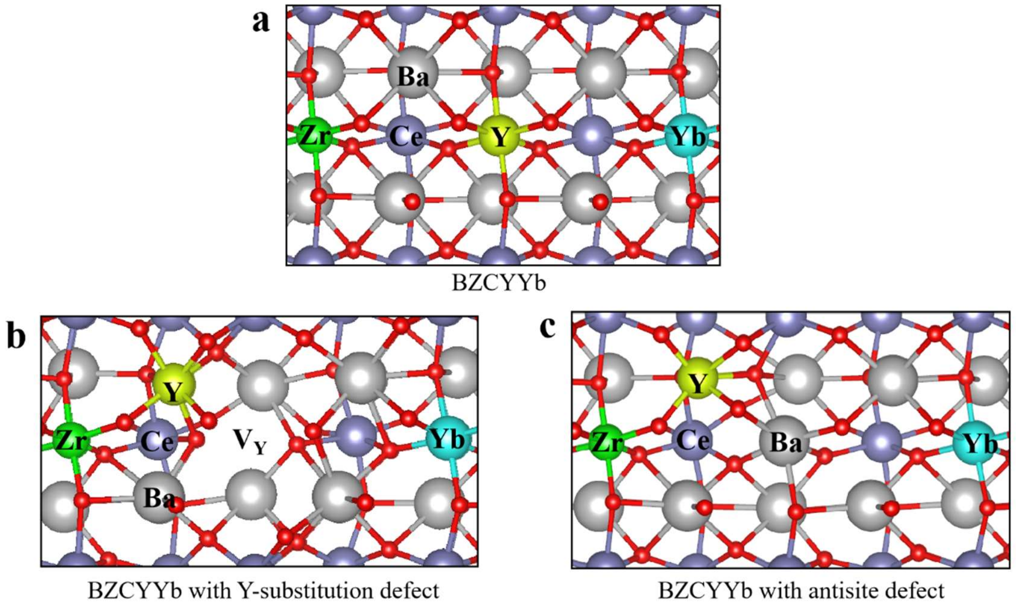

Supplementary Figure 41. The optimized structural models of (a) BZCYYb, (b) BZCYYb with Y-substitution defect, and (c) BZCYYb with antisite defect.

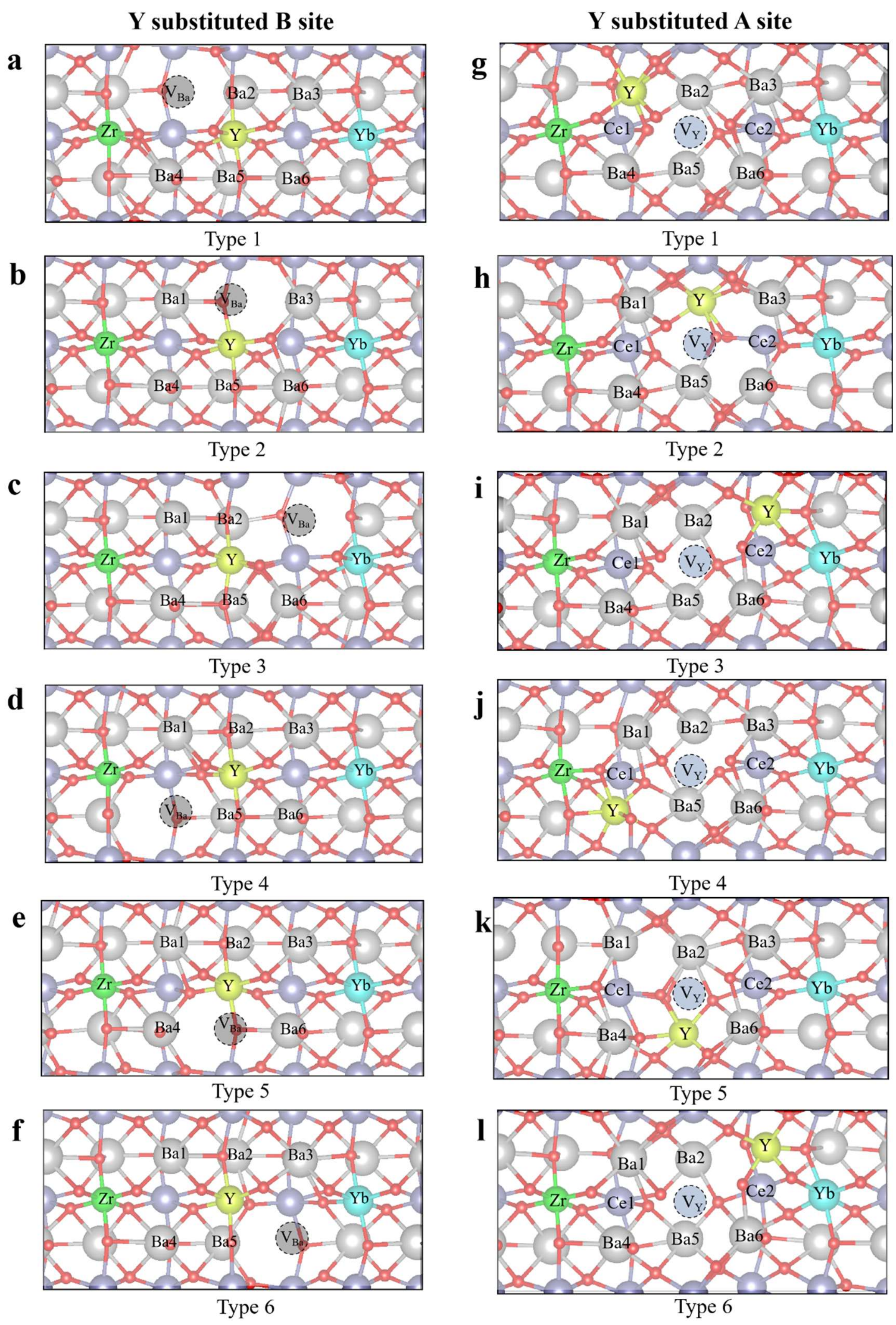

Supplementary Figure 42. Possible configurations for Y substituted B-site with Ba vacancy (a-f) and Y substituted A-site with Y vacancy (g-l) in BZCYYb.

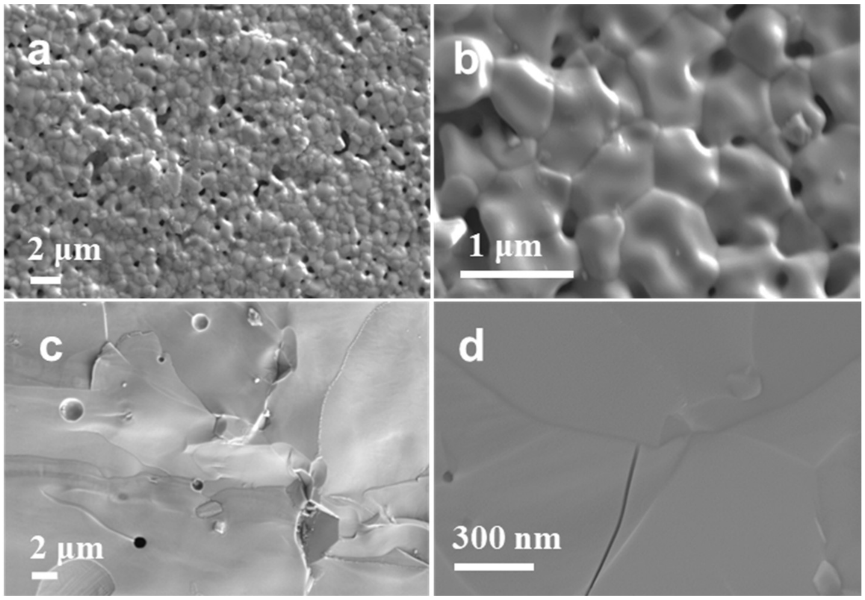

Supplementary Figure 43. a, b Surface and **c, d** cross-sectional SEM images of BZCYYb-SSR membrane sintered at 1450 ℃.

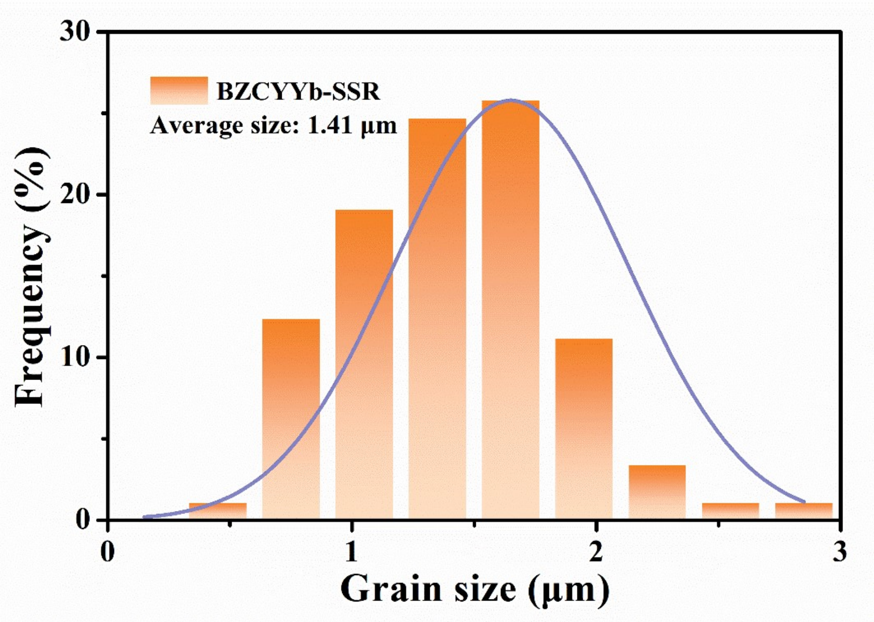

Supplementary Figure 44. Grain size distribution of BZCYYb-SSR electrolyte membrane sintered at 1450 °C.

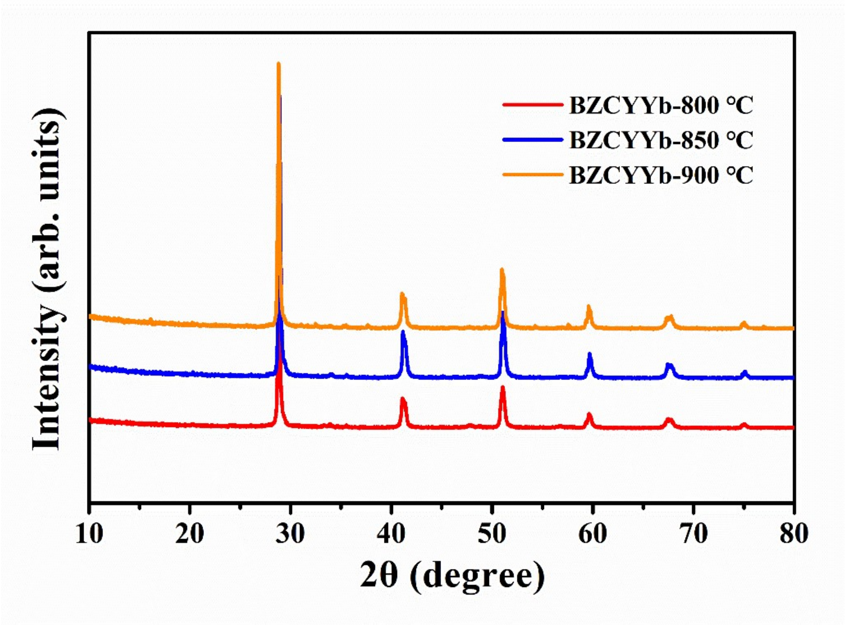

Supplementary Figure 45. XRD patterns of BZCYYb-MSS calcined at different temperatures in air for 5 h.

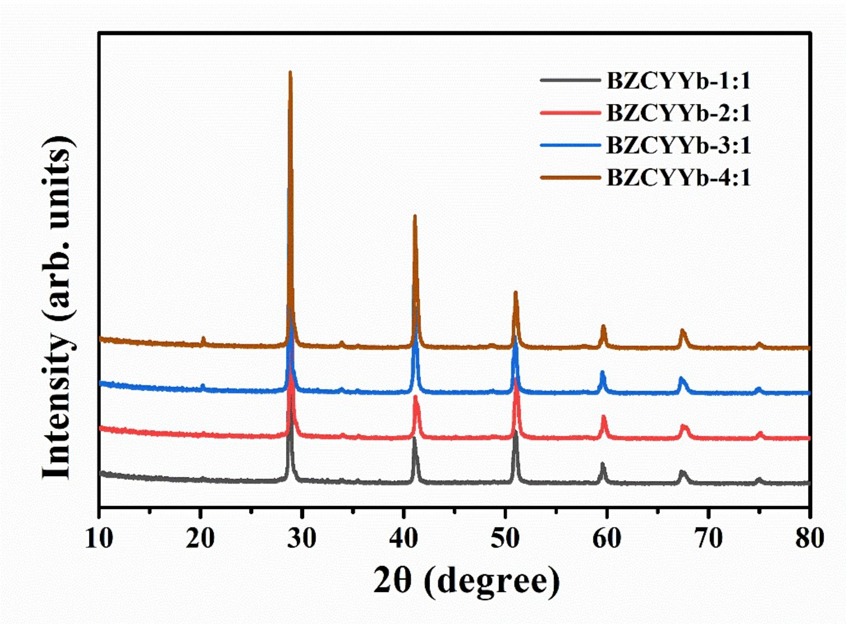

Supplementary Figure 46. XRD patterns of BZCYYb prepared by MSS method with different mass ratio of salt-to-nitrate at 850 °C in air.

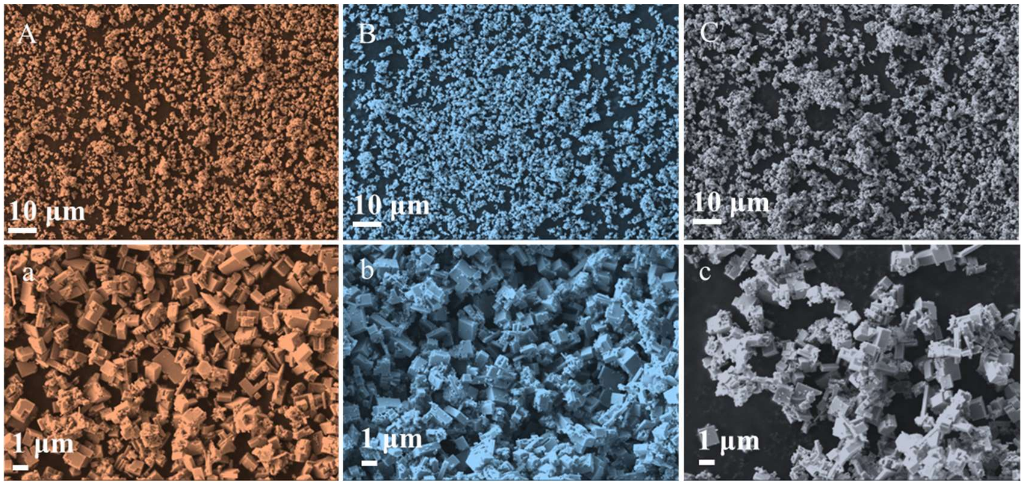

Supplementary Figure 47. SEM images of BZCYYb-MSS samples synthesized in different mass ratio of salt-to-nitrate. **(A, a)** 1：1. **(B, b)** 3：1. **(C, c)** 4：1.

Supplementary Discussion: Crystal growth of samples in the molten salt liquid depends on several factors, especially the mass ratio of molten salt to metal salt. When the ratio was 1：1, the particles were unevenly dispersed and slightly agglomerated. As the proportion of salt further increased to 3：1 and 4：1, particles agglomerated significantly and became irregular. The above results show that molten salt plays an important role in synthesis process, and excessive or insufficient is not conducive to morphology control.

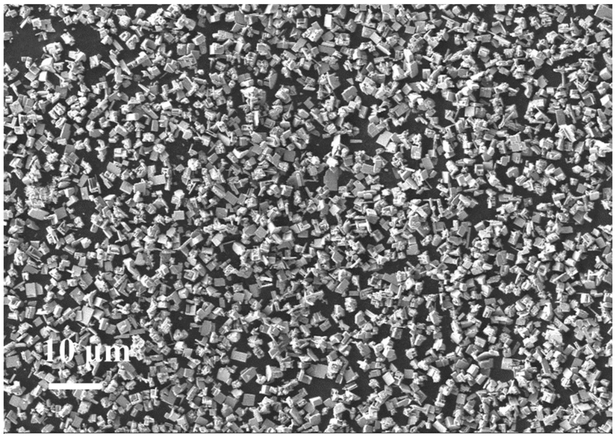

Supplementary Figure 48. SEM image of BZCYYb-MSS sample synthesized with the 2:1 salt-to-nitrate mass ratio.

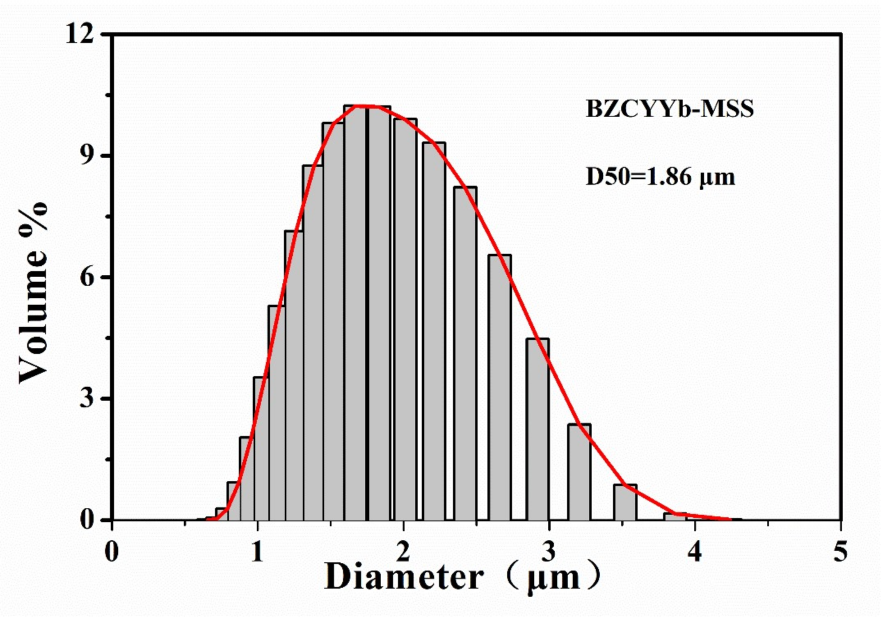

Supplementary Figure 49. Particle size analysis of BZCYYb-MSS.

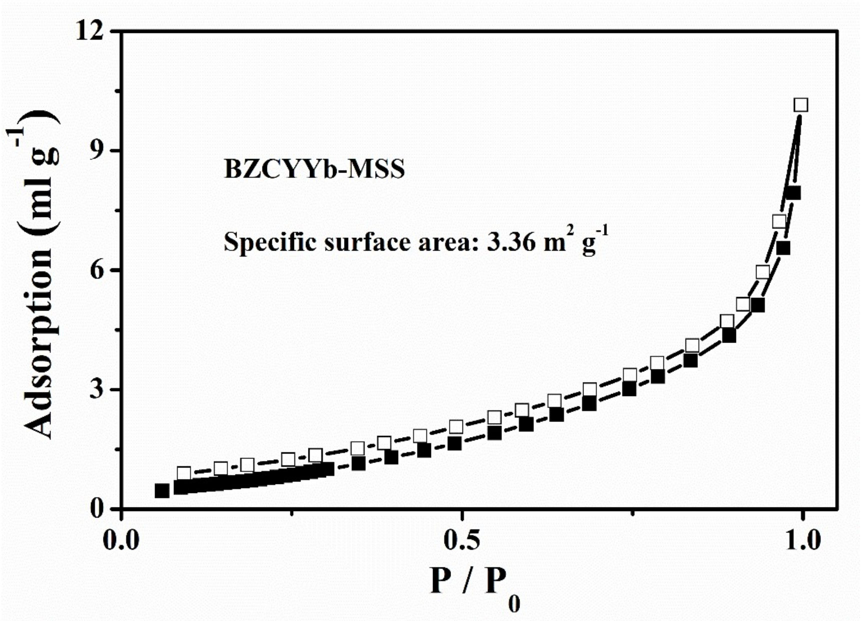

Supplementary Figure 50. The N_2 adsorption-desorption isotherm of pristine BZCYYb-MSS powder.

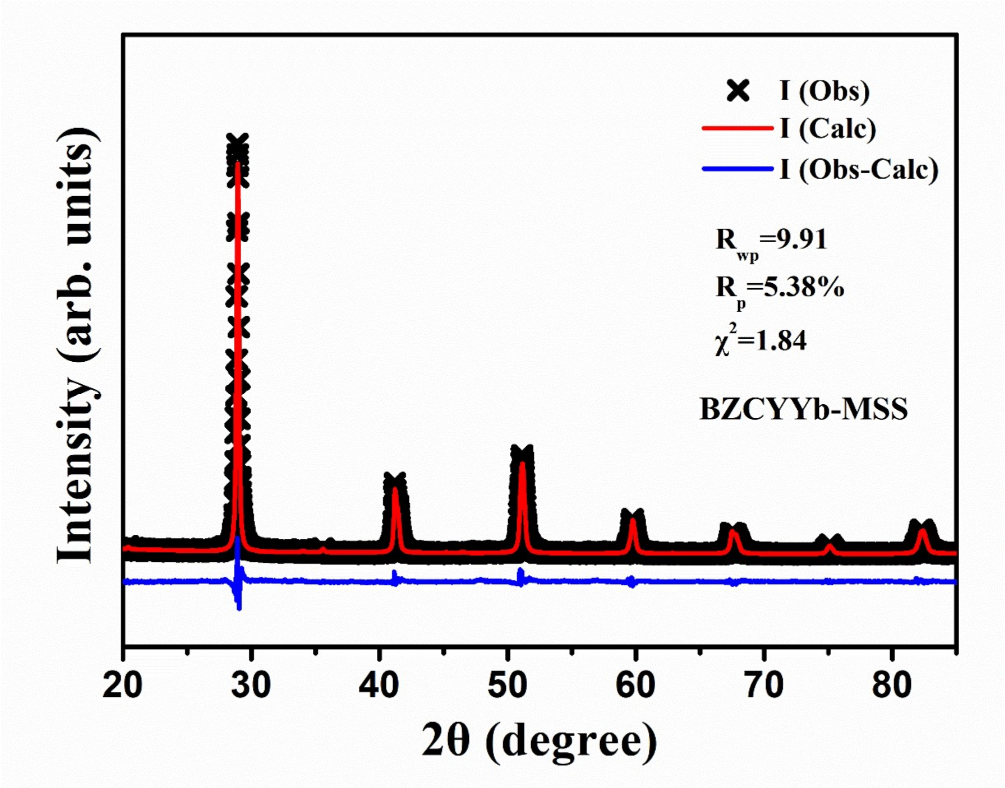

Supplementary Figure 51. Rietveld refinement plots of the XRD pattern of the pristine BZCYYb-MSS sample.

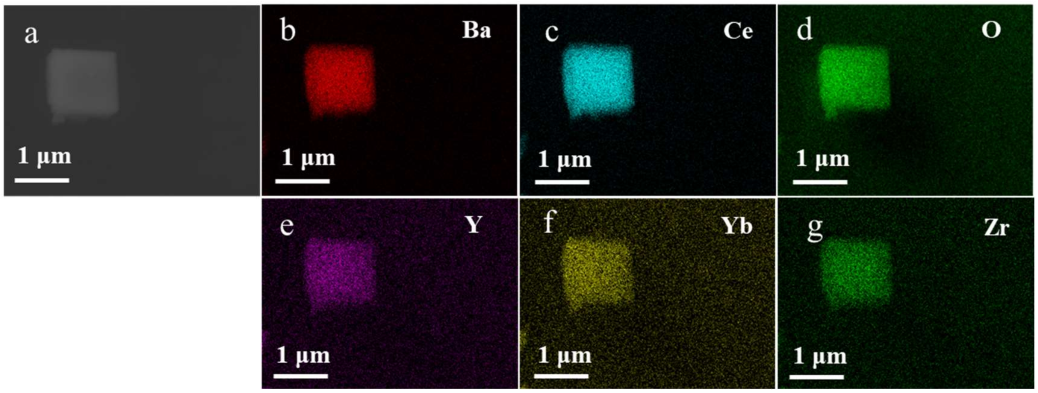

Supplementary Figure 52. SEM image of BZCYYb-MSS single-particle (a) and corresponding EDS mapping of Ba (b), Ce (c), O (d), Y (e), Yb (f), Zr (g).

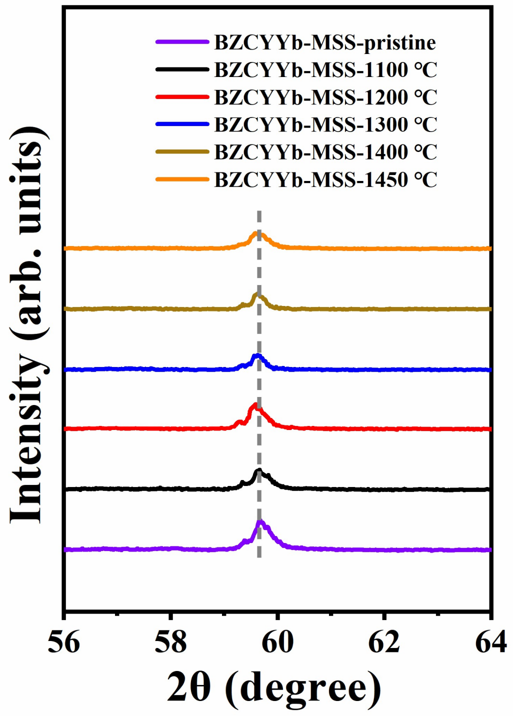

Supplementary Figure 53. Magnified XRD patterns of BZCYYb-MSS pristine sample and after quenching at different temperatures.

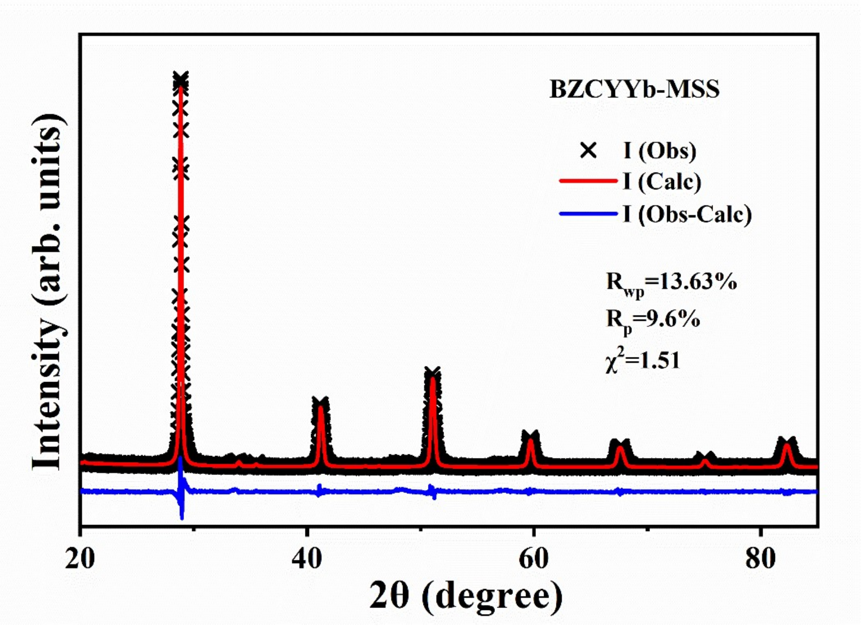

Supplementary Figure 54. Rietveld refinement plots of the XRD pattern of BZCYYb-MSS quenched at 1300 ℃.

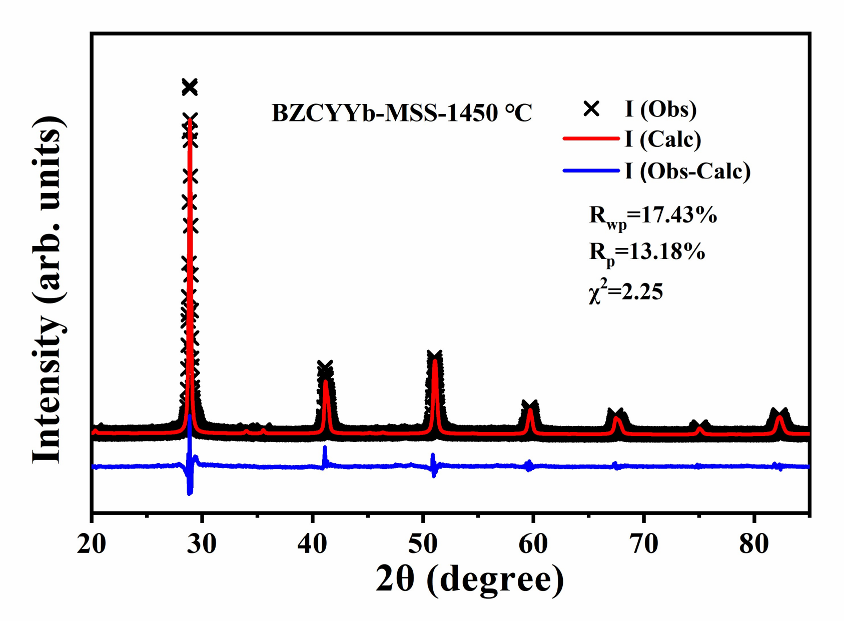

Supplementary Figure 55. Rietveld refinement plots of the XRD pattern of BZCYYb-MSS quenched at 1450 °C.

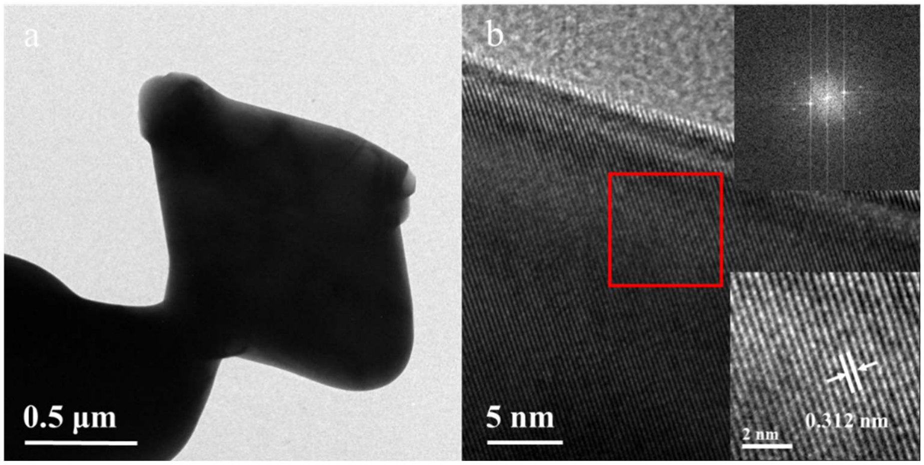

Supplementary Figure 56. a, b TEM images of BZCYYb-MSS powder quenched at 1300 ℃. Inset images are the corresponding fast Fourier transform (FFT) pattern of the area in the red box.

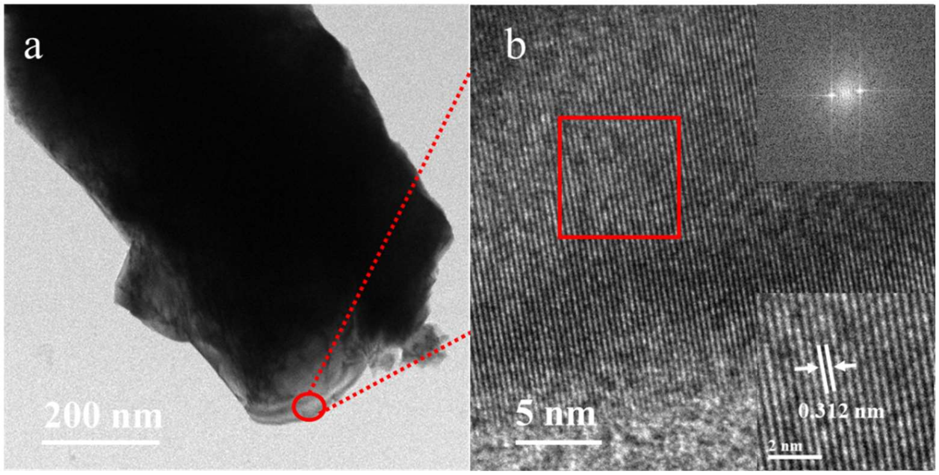

Supplementary Figure 57. a, b TEM images of BZCYYb-MSS powder quenched at 1450 °C. Inset images are the corresponding fast Fourier transform (FFT) pattern of the area in the red box.

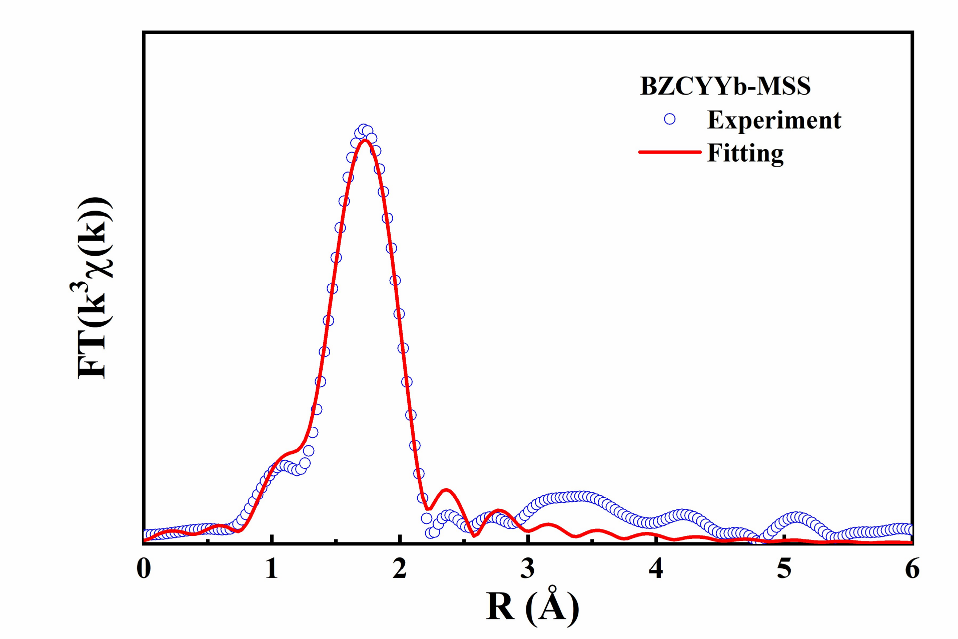

Supplementary Figure 58. Fourier-transformed EXAFS data measured at the Y *K*-edge and its fitting curve for BZCYYb-MSS.

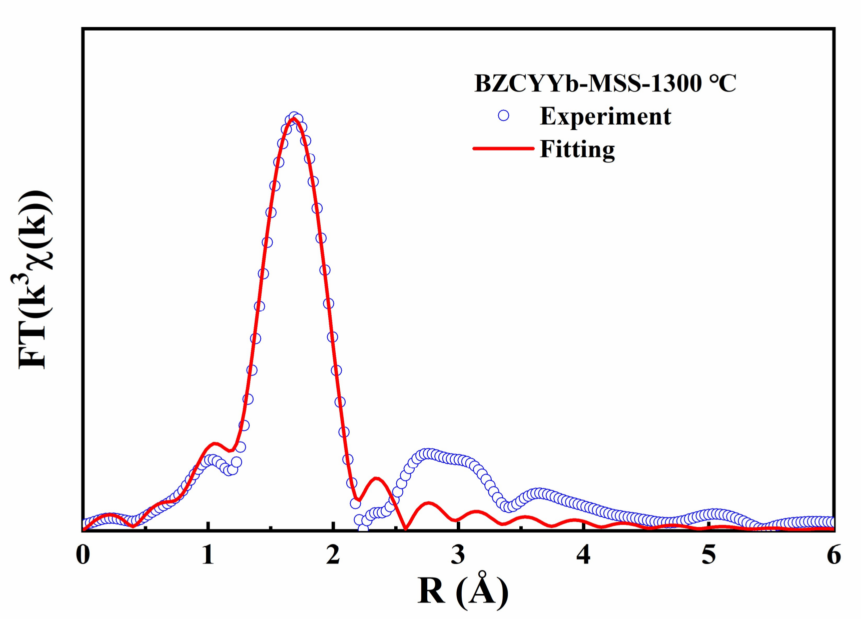

Supplementary Figure 59. Fourier-transformed EXAFS data measured at the Y *K*-edge and its fitting curve for BZCYYb-MSS after quenching at 1300 °C.

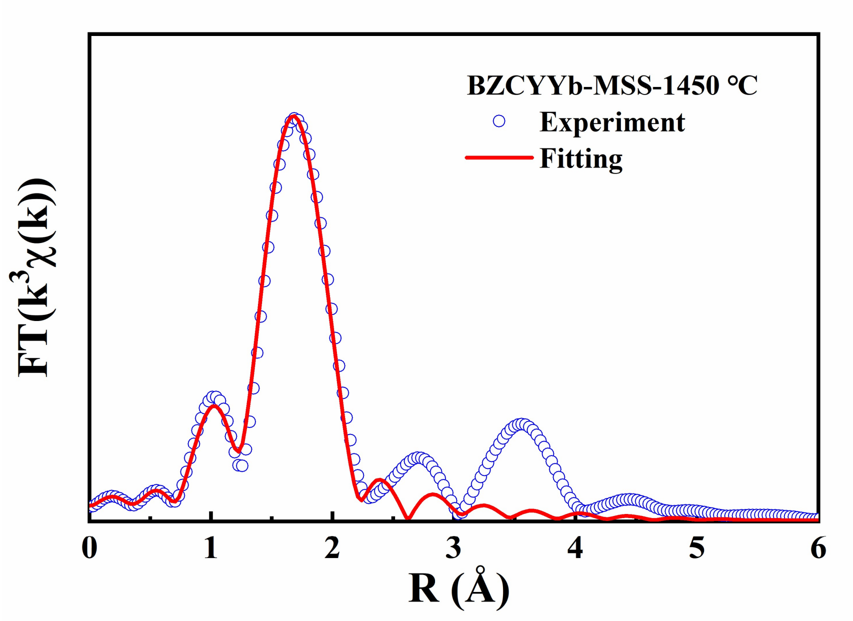

Supplementary Figure 60. Fourier-transformed EXAFS data measured at the Y *K*-edge and its fitting curve for BZCYYb-MSS after quenching at 1450 °C.

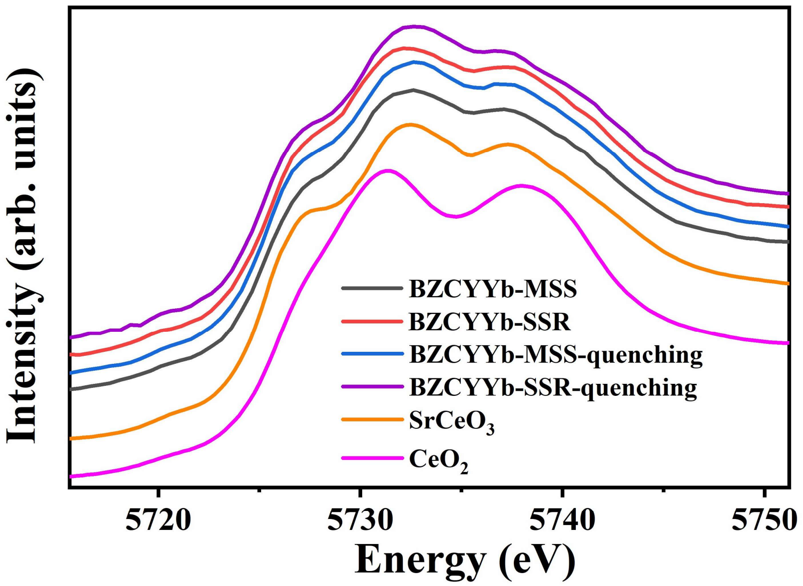

Supplementary Figure 61. The spectra of Ce L_3 edge XANES for BZCYYb-MSS and BZCYYb-SSR before and after quenching at 1300 °C.

Supplementary Discussion: To investigate the possibility of Ce migration, X-ray absorption near-edge structure (XANES) analysis was performed at the Ce L_3 edge of all BZCYYb samples as shown in **Supplementary Figure 61,** along with reference CeO_2 with local 8-fold coordination and $SrCeO_3$ with local 6-fold coordination. All feature and energy position of the Ce-L_3 XANES spectrum of BZCYYb before and after quenching at 1300 °C are almost same to those of $SrCeO_3$, confirming that Ce exists in a Ce^{4+} state with local octahedral coordination in all BZCYYb samples and exists in the B site of perovskite. The splitting of each peak at the Ce-L_3 edge due to 5d crystal field slitting reduces with increase in coordination number from CeO_6 in $SrCeO_3$ to CeO_8 in CeO_2[1], and will be further narrower at A site for CeO_{12}. Therefore, for both BZCYYb-MSS and BZCYYb-SSR samples, we can exclude meaningful mount of Ce ions at the A-site.

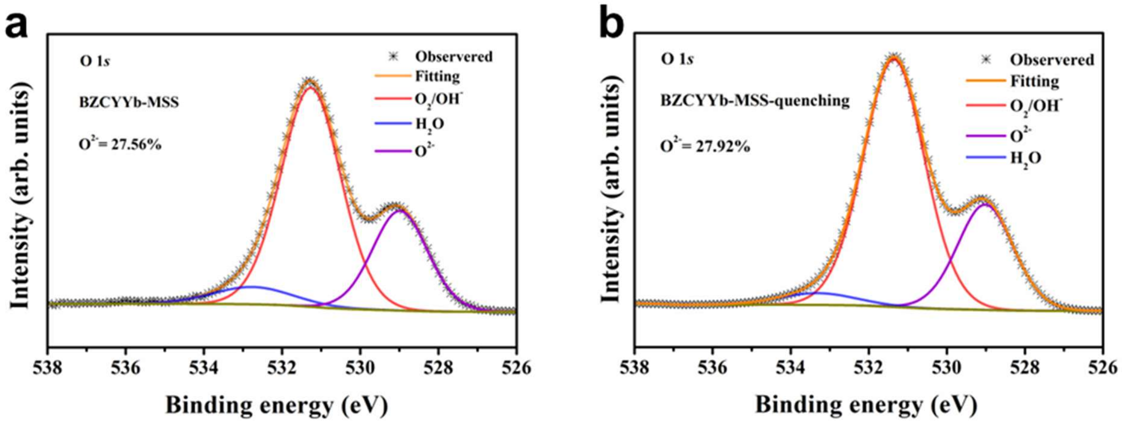

Supplementary Figure 62. a, b The XPS spectra of O 1*s* for BZCYYb-MSS before and after quenching.

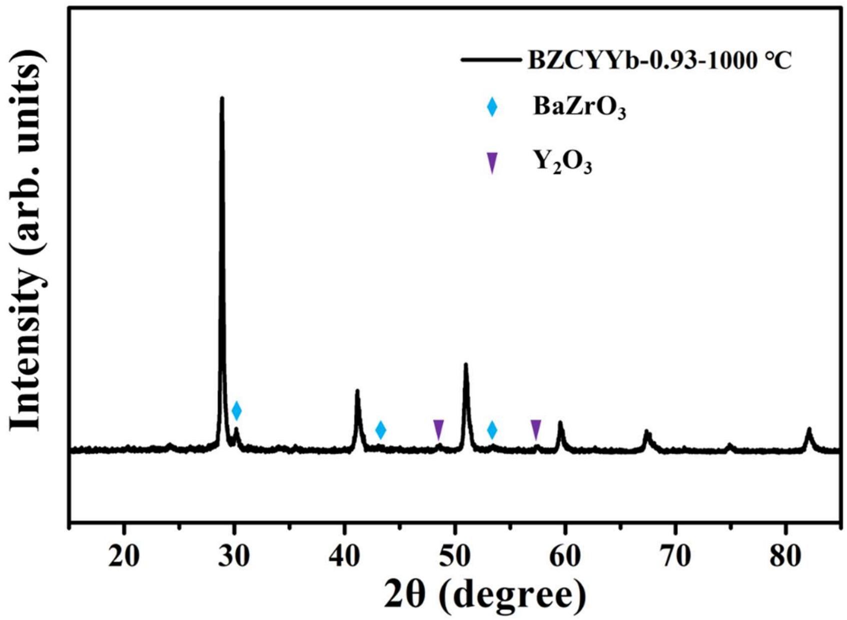

Supplementary Figure 63. XRD pattern of BZCYYb-0.93-SSR synthesized at 1000 ℃.

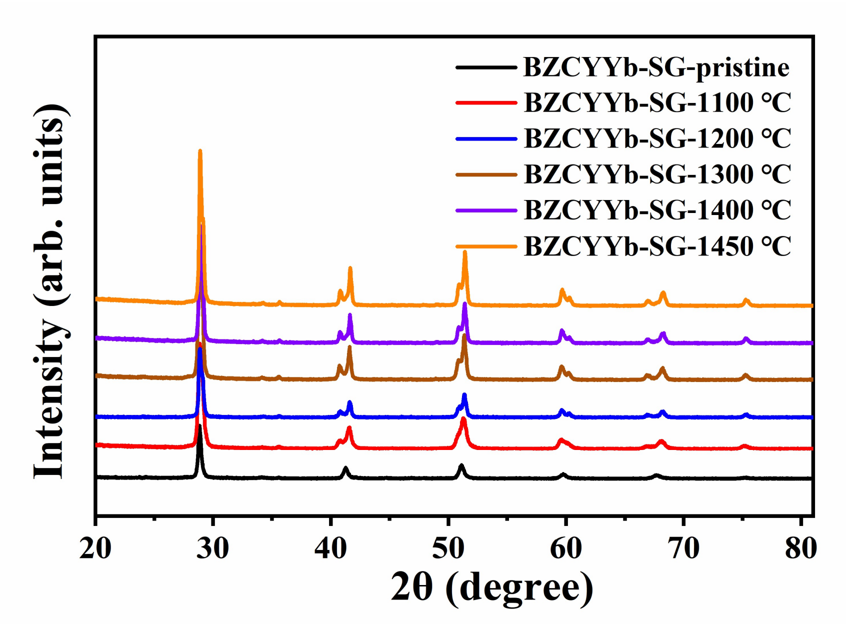

Supplementary Figure 64. XRD patterns of pristine BZCYYb-SG sample and after quenching at different temperatures.

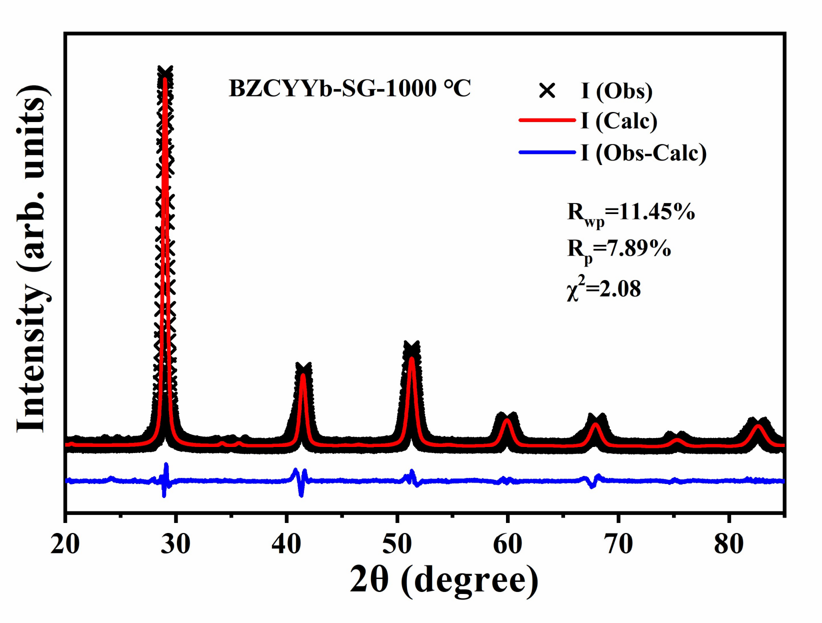

Supplementary Figure 65. Rietveld refinement plots of the XRD pattern of the pristine BZCYYb-SG sample.

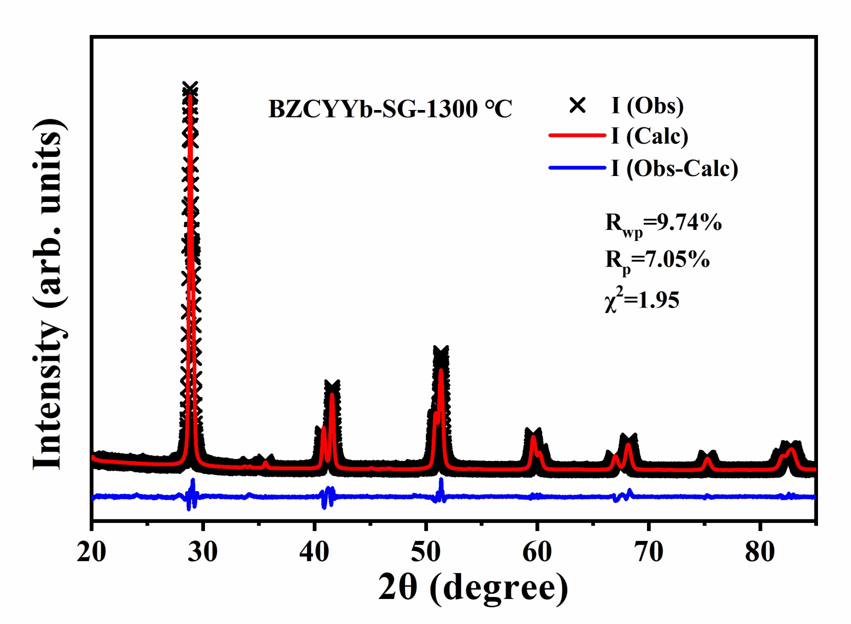

Supplementary Figure 66. Rietveld refinement plots of the XRD pattern of BZCYYb-SG quenched at 1300 °C.

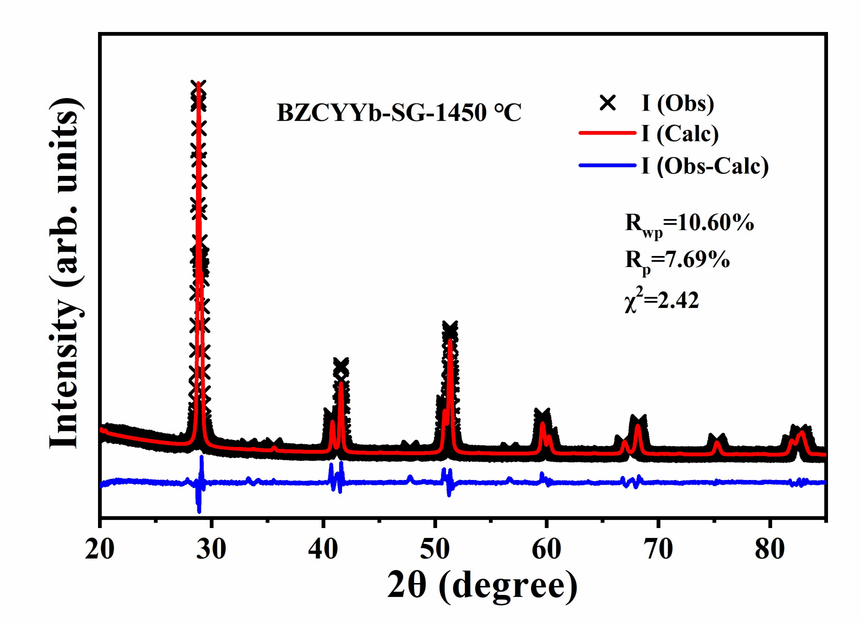

Supplementary Figure 67. Rietveld refinement plots of the XRD pattern of BZCYYb-SG quenched at 1450 °C.

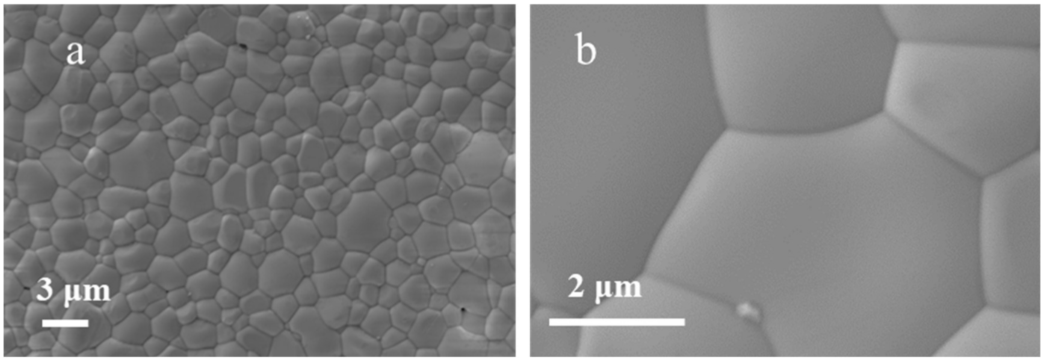

Supplementary Figure 68. a, b Surface SEM images of BZCYYb-MSS membrane sintered at 1450 ℃.

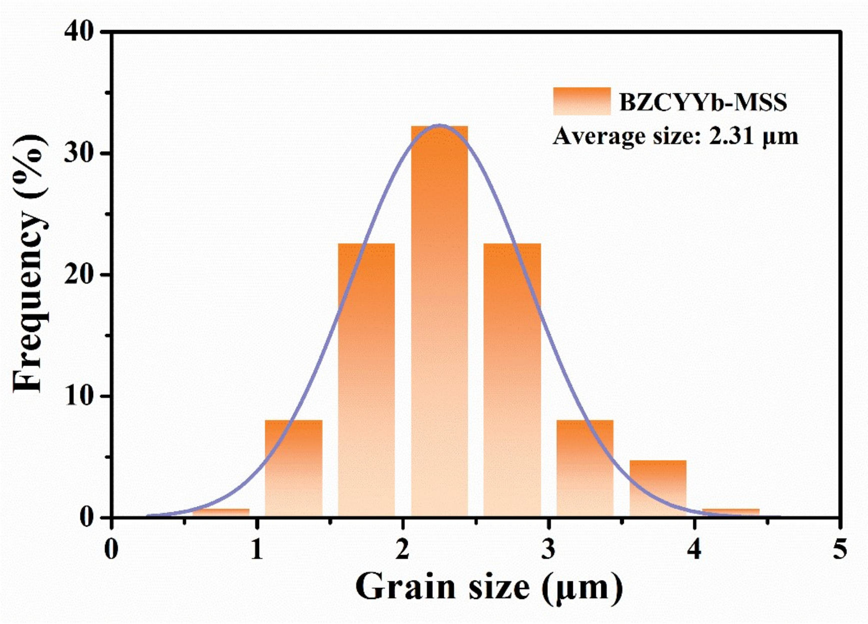

Supplementary Figure 69. Grain size distribution of BZCYYb-MSS electrolyte membrane sintered at 1450 ℃.

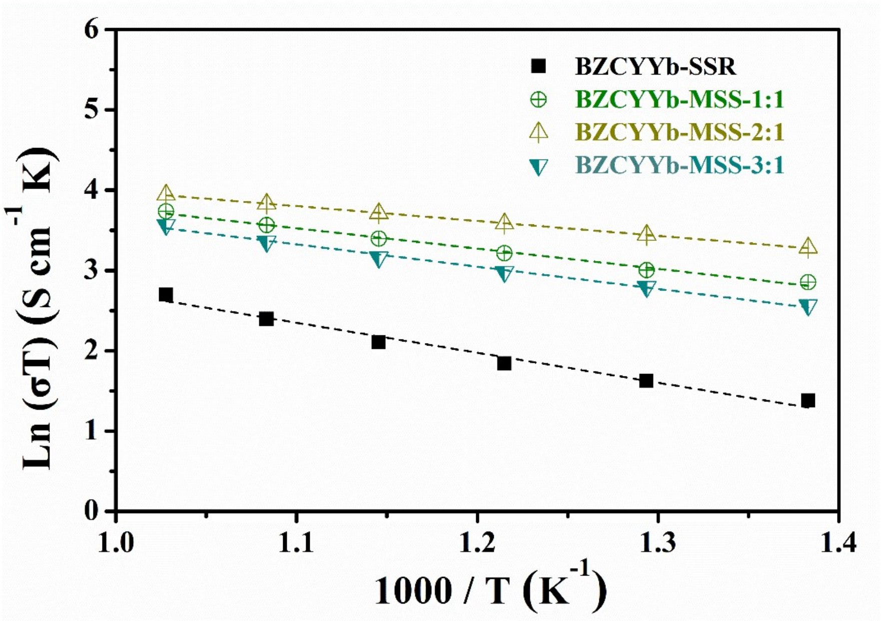

Supplementary Figure 70. Arrhenius plots of BZCYYb-MSS and BZCYYb-SSR electrolytes.

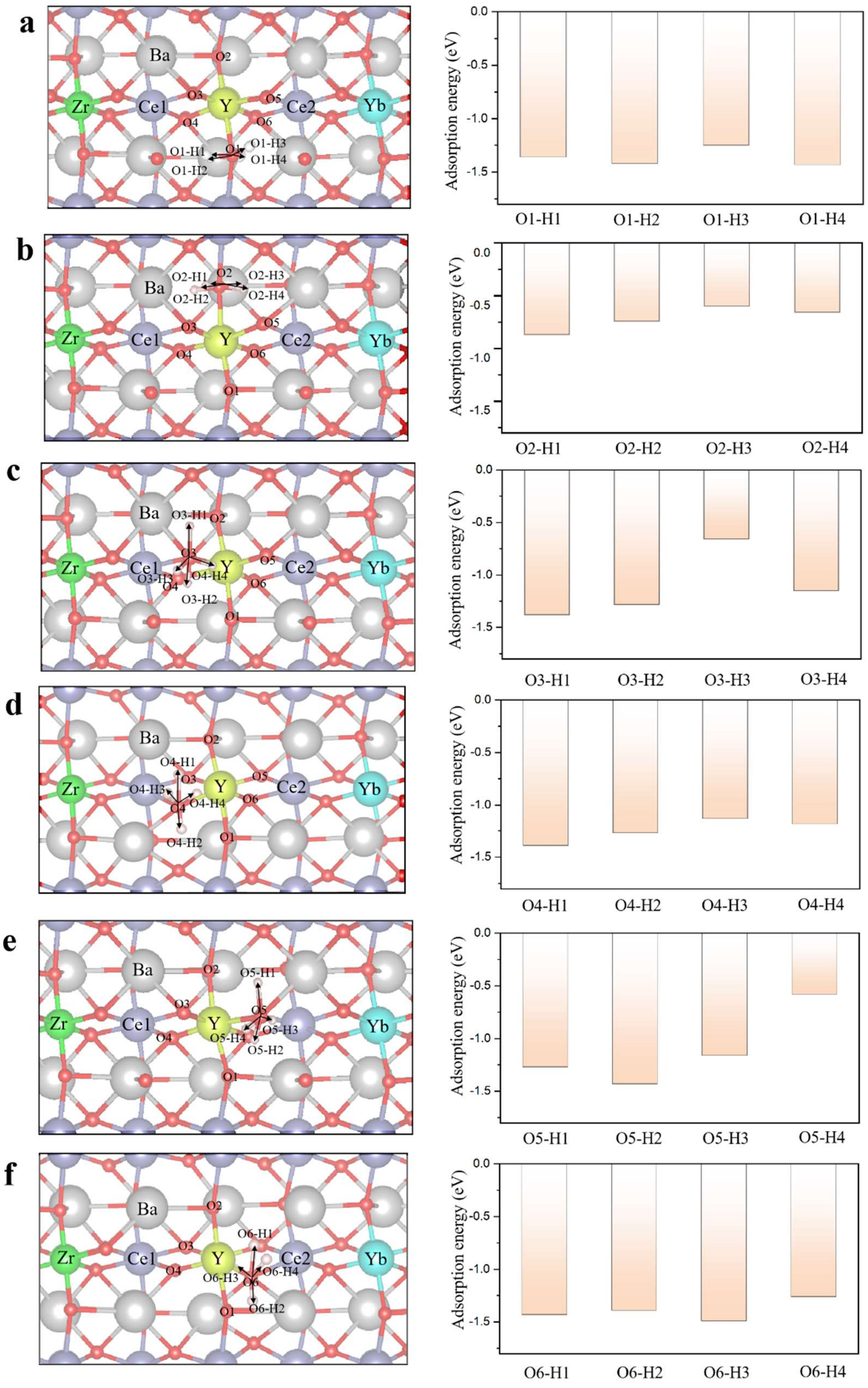

Supplementary Figure 71. Illustrations of proton positions along different orientations and corresponding proton adsorption energies in BZCYYb for Y substituted B-site.

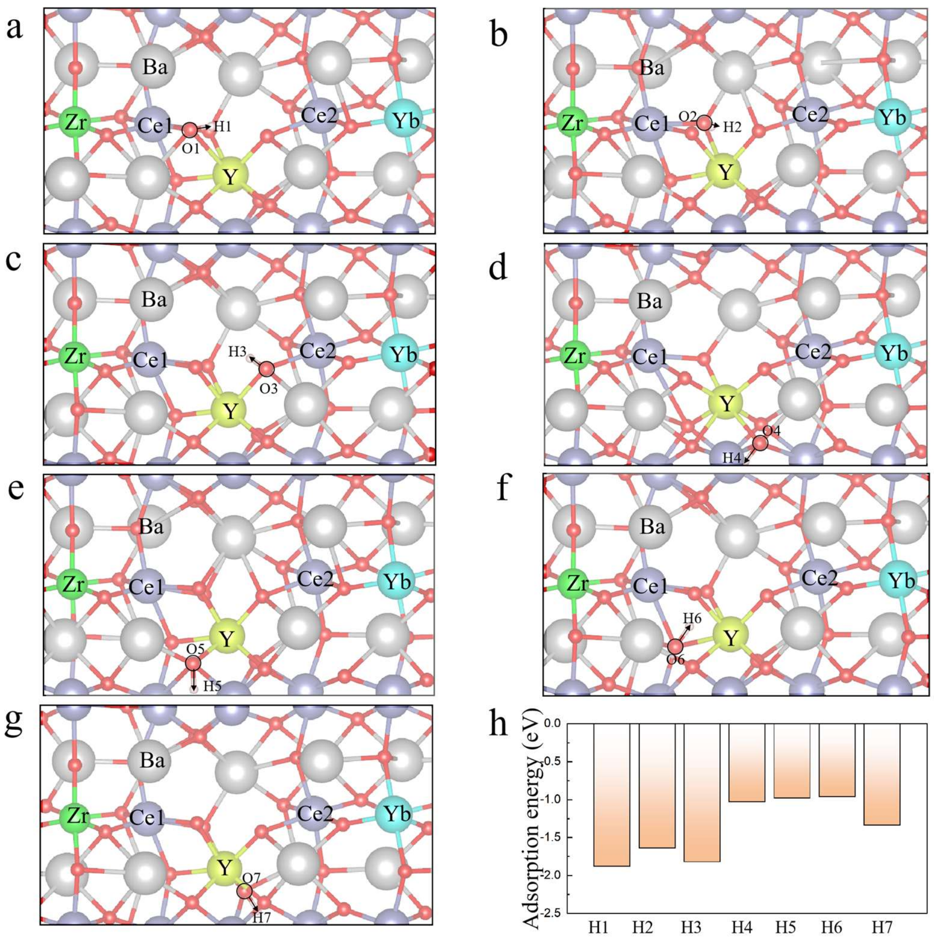

Supplementary Figure 72. Illustrations of proton positions along different orientations and corresponding proton adsorption energies in B site in BZCYYb for Y substituted A-site.

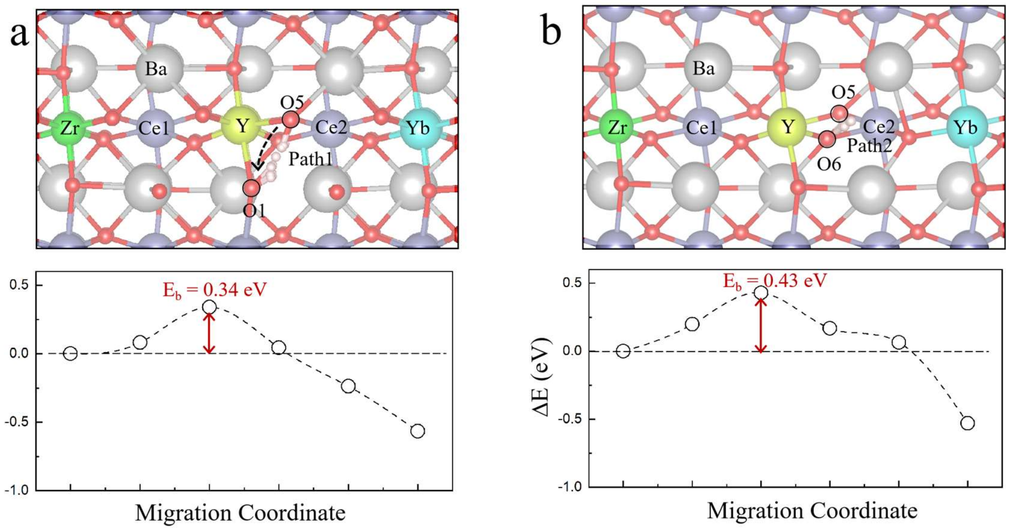

Supplementary Figure 73. **a, b** Diffusion path of proton between adjacent lattice oxygen sites and corresponding migration energy barriers for Y substituted B-site in BZCYYb.

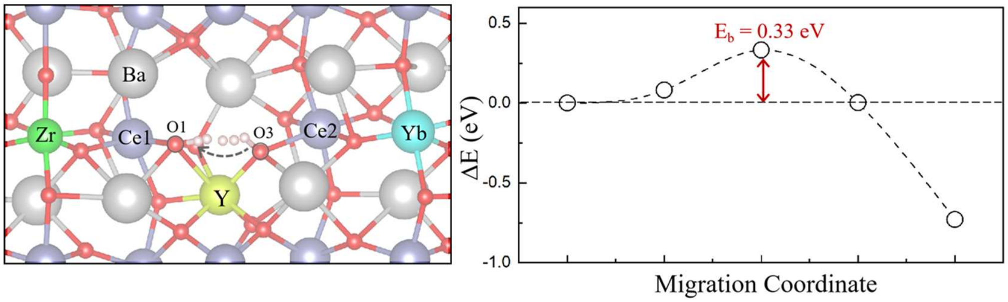

Supplementary Figure 74. Diffusion path of proton between adjacent lattice oxygen sites and corresponding migration energy barriers for Y substituted A-site in BZCYYb.

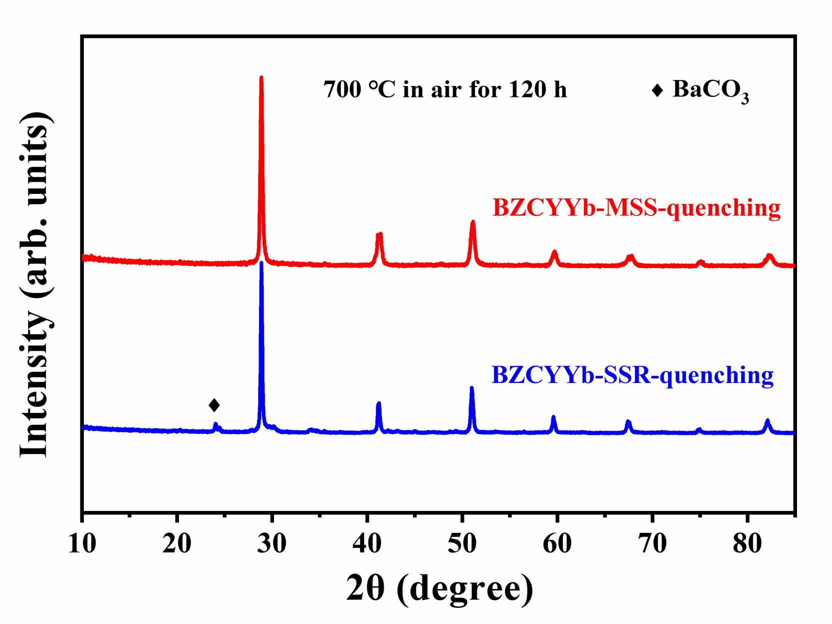

Supplementary Figure 75. XRD patterns of quenched BZCYYb-SSR and quenched BZCYYb-MSS samples calcined at 700 °C for 120 hours in air.

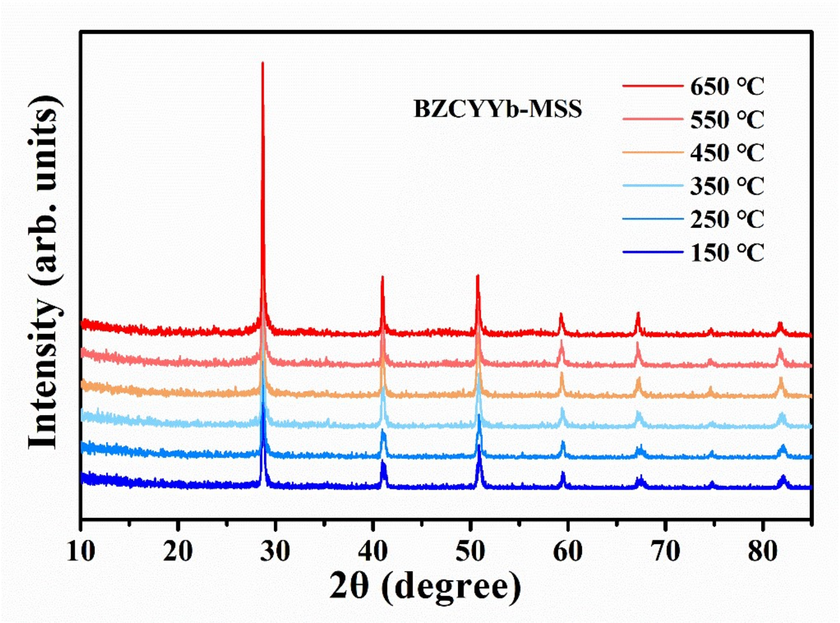

Supplementary Figure 76. In situ HT-XRD patterns for BZCYYb-MSS in 5% CO_2 balanced argon from 150 to 650 °C.

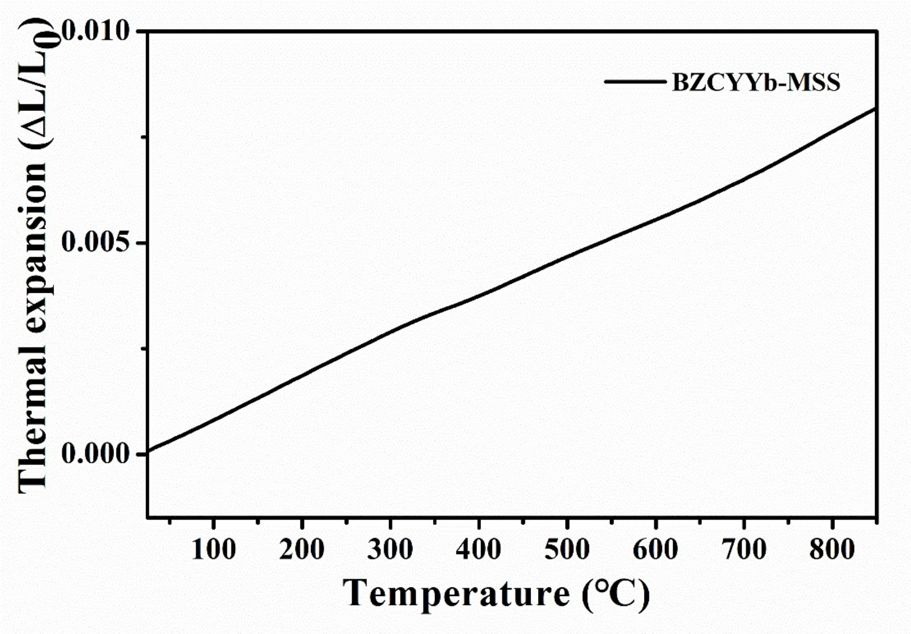

Supplementary Figure 77. Thermal expansion curve of BZCYYb-MSS.

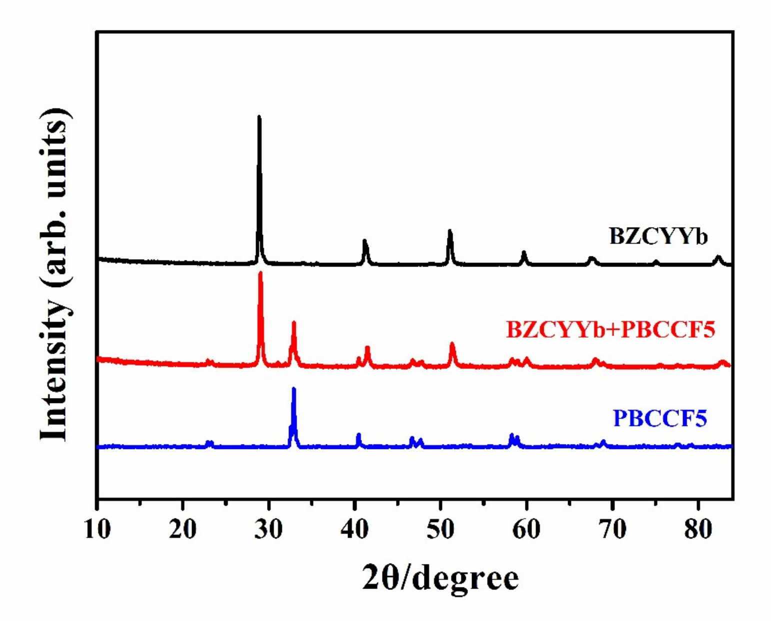

Supplementary Figure 78. The XRD measurement of chemical compatibility between BZCYYb-MSS electrolyte and PBCCF5 electrode. The mixed powder (1:1 wt.%) was calcined at 1000°C in air for 5 h.

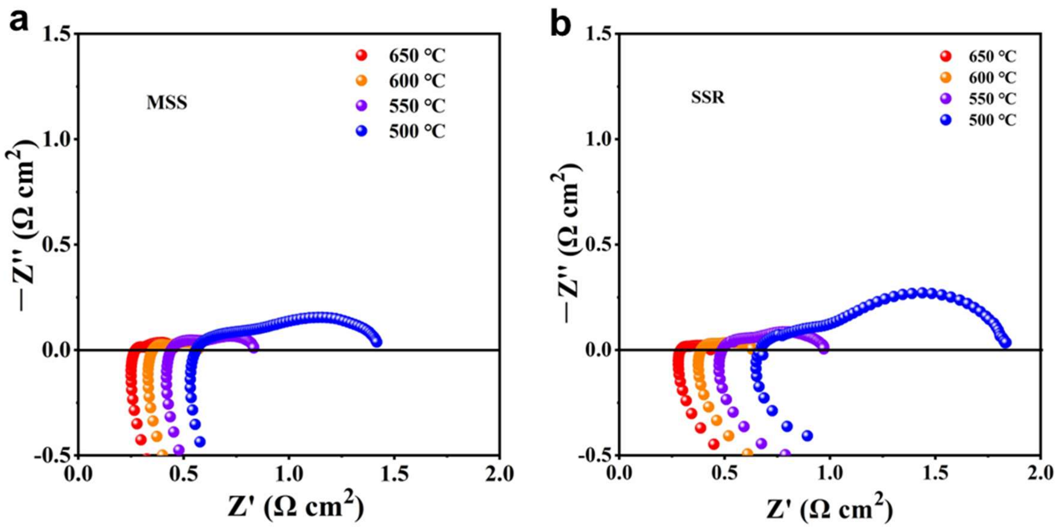

Supplementary Figure 79. Electrochemical impedance spectra of the cell with BZCYYb-MSS (a) and BZCYYb-SSR (b) as electrolyte from 500 to 650 °C.

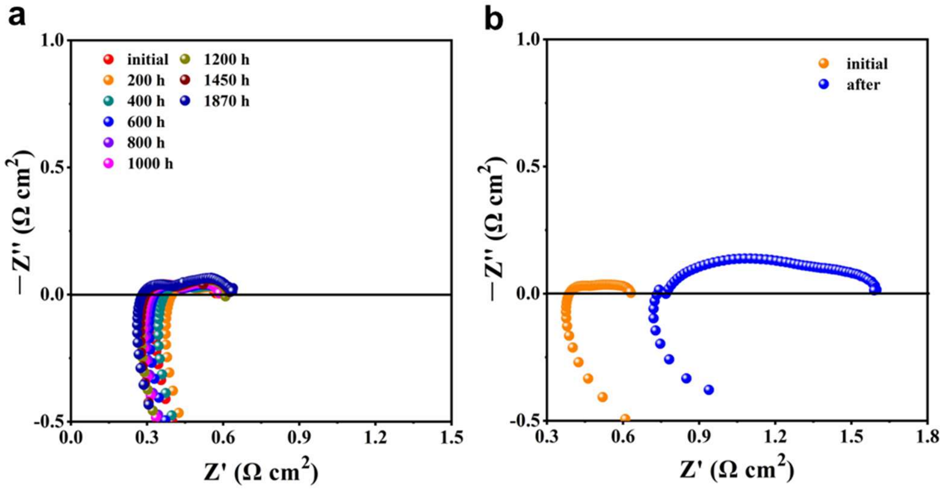

Supplementary Figure 80. Impedance stability of the cell with BZCYYb-MSS (a) and BZCYYb-SSR (b) as electrolyte at 600 ℃.

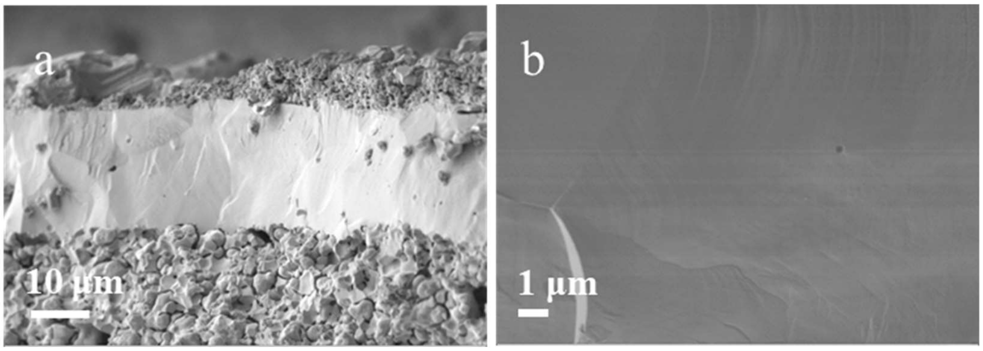

Supplementary Figure 81. a Cross-sectional SEM image of BZCYYb-MSS-based single cell after the 2000 h long-term stability test. **b** High-magnification SEM image of BZCYYb-MSS electrolyte.

Supplementary Discussion: As shown, cross-section SEM images of the respective three layers of BZCYYb-MSS based single cell, including a Ni-BZCYYb anode, a dense BZCYYb electrolyte layer, and a PBCCF5 cathode layer. And the three layers were adhered well with no cracks or delamination after 2000 h long-term fuel cell operation. The high density BZCYYb electrolyte membrane confirmed that the BZCYYb electrolyte prepared by molten salt synthesis method did not have obvious grain boundaries and porous structure.

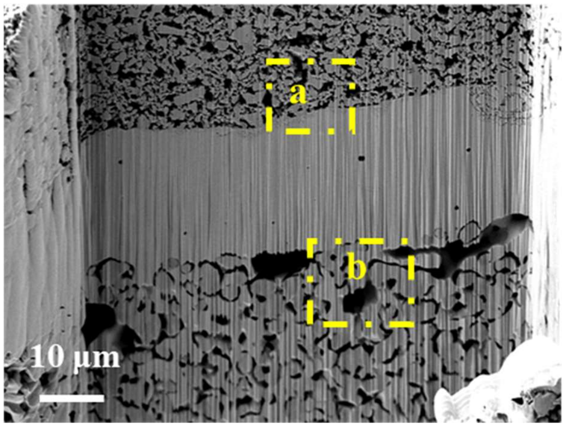

Supplementary Figure 82. FIB-SEM image of BZCYYb-MSS-based single cell with super dense electrolyte layer and two porous electrode layers after the 2000 h long-term stability test.

Supplementary Discussion: FIB-SEM and EDS mapping of the BZCYYb-MSS-based single cell after the long-term stability test were also investigated, as shown in **Supplementary Figures 82-84**. The FIB-SEM images illustrate that both the anode and cathode are well combined with the electrolyte without any cracks and precipitates at the interfaces after the stability test. The EDS mapping clearly shows the uniform distribution of elements in the three layers, which indicates that no chemical reactions and element diffusion occurred during the stability test.

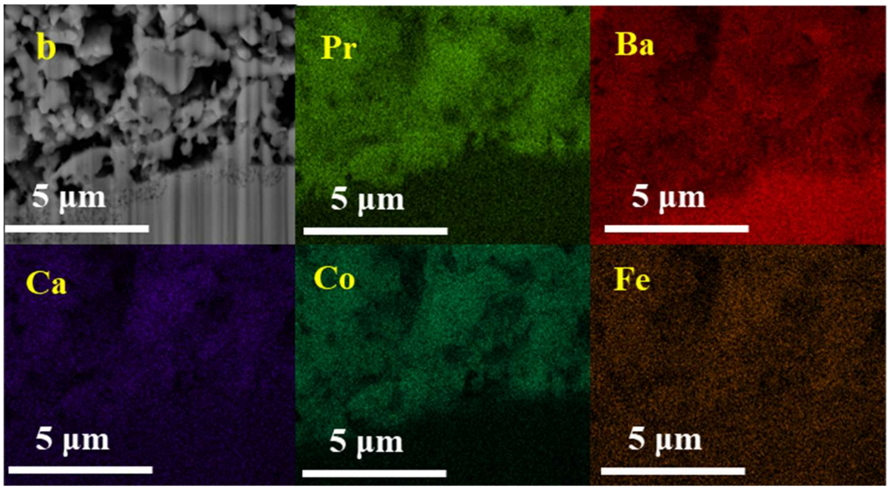

Supplementary Figure 83. SEM image of PBCCFe5 cathode and the corresponding EDS mapping of Pr, Ba, Ca, Co, Fe near the interface at position a in **supplementary Figure 82**.

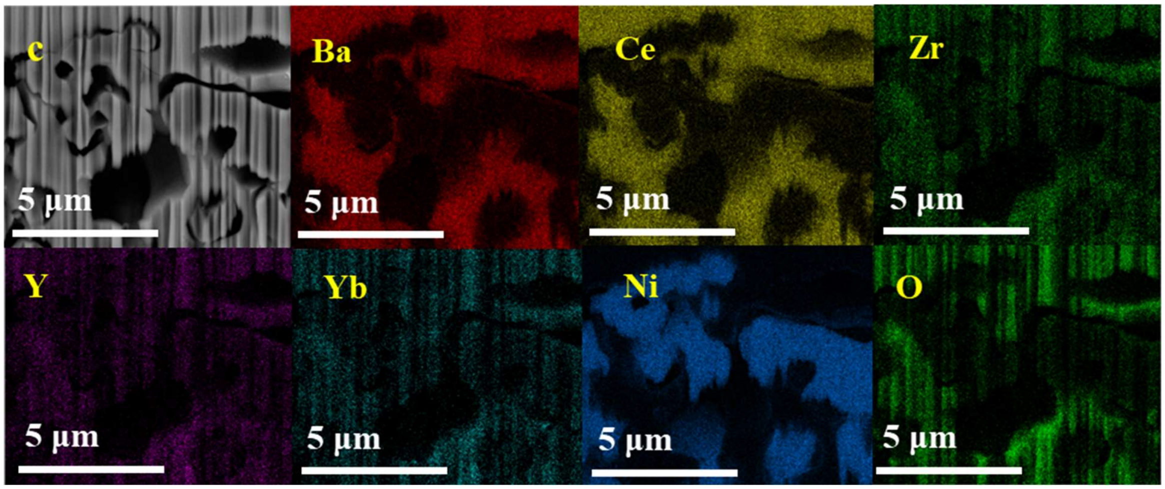

Supplementary Figure 84. SEM image of Ni-BZCYYb anode and the corresponding EDS mapping of Ba, Ce, Zr, Y, Yb near the interface at position b in **supplementary Figure 82**.

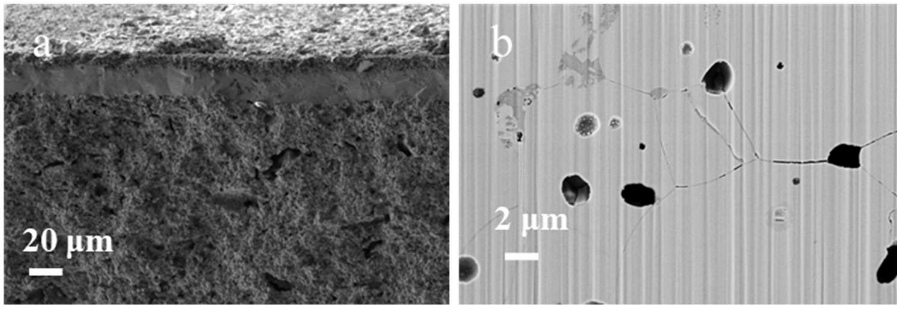

Supplementary Figure 85. a Cross-sectional SEM image of the BZCYYb-SSR-based single cell after stability test. **b** FIB-SEM image of BZCYYb-SSR electrolyte.

Supplementary Tables

Supplementary Table 1. Cation compositions of BZCYYb-MSS and BZCYYb-SSR powders from HR-ICP-MS.

	Ba	Ce	Zr	Y	Yb
	Atomic ratio over (Ce + Zr +Y + Yb)				
BZCYYb-MSS	0.93	0.71	0.08	0.11	0.10
BZCYYb-SSR	1.01	0.71	0.10	0.10	0.10

Supplementary Table 2. Cation compositions of BZCYYb-MSS and BZCYYb-SSR powders from EDS mapping.

	Ba	Ce	Zr	Y	Yb
	Atomic ratio over (Ce + Zr + Y + Yb)				
BZCYYb-MSS	0.93	0.71	0.10	0.10	0.10
BZCYYb-SSR	1.00	0.70	0.10	0.10	0.10

Supplementary Table 3. XRD refinement parameters of BZCYYb samples before and after quenching.

Samples	R_p (%)	R_{wp} (%)	R_{exp} (%)	Space group	a (Å)	b (Å)	c (Å)	Volume (Å^3)
BZCYYb-MSS	5.38	9.91	6.04	*Imma*	6.21	8.74	6.22	338.0
BZCYYb-MSS-1300 ℃	9.60	13.63	9.03	*Imma*	6.21	8.74	6.21	337.5
BZCYYb-MSS-1450 ℃	13.18	17.43	7.76	*Imma*	6.21	8.73	6.21	337.1
BZCYYb-SSR	7.49	10.86	5.56	*Imma*	6.21	8.81	6.22	340.3
BZCYYb-SSR-1300 ℃	4.79	6.37	3.72	*Imma*	6.15	8.87	6.15	335.8
BZCYYb-SSR-1450 ℃	8.03	11.73	4.25	*Imma*	6.15	8.85	6.15	335.4
BZCYYb-0.98-SSR	11.23	15.63	13.39	*Imma*	6.21	8.81	6.21	339.6
BZCYYb-0.98-SSR-1300 ℃	8.21	11.20	5.42	*Imma*	6.15	8.86	6.15	335.5
BZCYYb-0.98-SSR-1450 ℃	7.46	10.52	5.19	*Imma*	6.15	8.84	6.15	335.2
BZCYYb-0.95-SSR	11.90	16.94	13.27	*Imma*	6.20	8.81	6.21	339.5
BZCYYb-0.95-SSR-1300 ℃	6.56	9.91	5.53	*Imma*	6.15	8.87	6.15	335.6
BZCYYb-0.95-SSR-1450 ℃	7.94	11.30	5.69	*Imma*	6.15	8.84	6.15	335.2
BZCYYb-0.93-SSR	12.06	17.25	14.05	*Imma*	6.17	8.81	6.21	337.4
BZCYYb-0.93-SSR-1300 ℃	10.07	12.60	4.50	*Imma*	6.15	8.71	6.21	335.5
BZCYYb-0.93-SSR-1450 ℃	8.40	11.96	4.93	*Imma*	6.15	8.84	6.16	335.3

Supplementary Table 4. XRD refinement parameters of BZCYYb-1.05-SSR samples before and after quenching.

Samples	R_p (%)	R_{wp} (%)	R_{exp} (%)	Space group	a (Å)	b (Å)	c (Å)	Volume (Å^3)
BZCYYb-1.05-SSR	7.84	10.59	5.68	*Imma*	6.22	8.80	6.22	340.2
BZCYYb-1.05-SSR-1300 °C	13.40	18.52	14.05	*Imma*	6.17	8.87	6.15	336.6
BZCYYb-1.05-SSR-1450 °C	6.65	8.96	4.55	*Imma*	6.16	8.87	6.15	336.2

Supplementary Table 5. The structural parameters of the samples derived from R-space fitting curves of EXAFS on Y *K*-edge.

Samples	bond type	CN*	R (Å)	σ^2 (10^{-3}Å^2)**	R factor
Y_2O_3	Y-O	6	2.29±0.02	4.3±1.3	0.001
BZCYYb-MSS	Y-O	6.3±0.7	2.31±0.01	6.8±1.5	0.015
BZCYYb-MSS-1300 ℃	Y-O	6.4±0.5	2.29±0.02	7±1.0	0.011
BZCYYb-MSS-1450 ℃	Y-O	6.4±0.6	2.29±0.01	6.6±1.3	0.014
BZCYYb-SSR-	Y-O	6.0±0.5	2.33±0.02	6.1±1.3	0.007
BZCYYb-SSR-1300 ℃	Y-O	6.6±0.8	2.27±0.02	7.3±0.6	0.004
BZCYYb-SSR-1450 ℃	Y-O	6.6±0.7	2.27±0.01	6.6±1.4	0.010

* CN: coordination number; S_0^2 was fixed to be 0.97 obtained from the fitting of Y_2O_3 reference.

** σ^2: Debye−Waller factors.

Supplementary Table 6. The total energy of different $BaZr_{0.1}Ce_{0.7}Y_{0.1}Yb_{0.1}O_3$ configurations obtained by DFT and DFT+*U* methods.

	DFT	DFT+*U*
Type 1	-414.67	-396.18
Type 2	-414.67	-396.18
Type 3	-414.67	-396.18
Type 4	-414.67	-396.18
Type 5	-414.66	-396.09
Type 6	-414.62	-396.09
Type 7	-414.65	-396.11
Type 8	/	-396.11
Type 9	/	-396.08
Type 10	/	-396.16

Supplementary Table 7. Configuration parameters of theoretical model of BZCYYb.

	Lattice parameter						
BZCYYb-theoretical	a (Å)	b (Å)	c (Å)	α (°)	β (°)	γ (°)	Volume ($Å^3$)
	19.42	9.1	6.39	90.3	90	90	1128.48
	M-O bond length (Å)						
	Ba-O7	Ba-O1	Ba-O2	Ba-O3	Ba-O4	Ba-O5	Ba-O6
	2.75	2.70	2.79	3.25	2.88	2.85	2.98

Supplementary Table 8. Configuration parameters of BZCYYb with Y-substitution defect, where Y occupies the Ba site, and the Y original position is empty.

BZCYYb-model 1	Lattice parameter						
	a (Å)	b (Å)	c (Å)	α (°)	β (°)	γ (°)	Volume (Å^3)
	19.45	9.08	6.34	91.2	90.1	89.8	1118.65
	M-O bond length (Å)						
	Y-O7	Y-O1	Y-O2	Y-O3	Y-O4	Y-O5	Y -O6
	2.39	2.45	2.52	2.47	2.33	2.33	2.38

Supplementary Table 9. Configuration parameters of BZCYYb with antisite defect, where Y occupies the Ba site and Ba occupies the Y site.

BZCYYb-model 2	Lattice parameter						
	a (Å)	b (Å)	c (Å)	α (°)	β (°)	γ (°)	Volume ($Å^3$)
	19.47	9.12	6.38	90.2	90.3	90.1	1132.69
	M-O bond length (Å)						
	Y-O7	Y -O1	Y-O2	Y-O3	Y-O4	Y-O5	Y-O6
	2.24	2.32	2.38	2.81	2.30	2.37	2.50

Supplementary Table 10. The total energies (eV) for different configurations of Y substituted B-site and A-site in BZCYYb.

	Type 1	Type 2	Type 3	Type 4	Type 5	Type 6
B site	-385.9749	-386.0016	-386.0950	-385.8773	-386.0586	-385.9062
A site	-385.4496	-385.8894	-385.6135	-385.9061	-386.1093	-385.6173

Supplementary Table 11. Cation compositions of BZCYYb-MSS and BZCYYb-SSR powders after quenching at different temperatures from HR-ICP-MS.

	Ba	Ce	Zr	Y	Yb
	Atomic ratio over (Ce + Zr +Y + Yb)				
BZCYYb-MSS-1300 ℃	0.92	0.71	0.09	0.11	0.10
BZCYYb-MSS-1450 ℃	0.92	0.72	0.08	0.10	0.09
BZCYYb-SSR-1300 ℃	0.97	0.72	0.09	0.10	0.10
BZCYYb-SSR-1450 ℃	0.95	0.71	0.09	0.10	0.10

Supplementary Table 12. XRD refinement parameters of BZCYYb-SG samples before and after quenching.

Samples	R_p (%)	R_{wp} (%)	R_{exp} (%)	Space group	a (Å)	b (Å)	c (Å)	Volume (Å^3)
BZCYYb-SG	7.89	11.45	5.50	*Imma*	6.21	8.79	6.25	341.0
BZCYYb-SG-1300 ℃	7.05	9.74	5.00	*Imma*	6.15	8.86	6.15	335.4
BZCYYb-SG-1450 ℃	7.69	10.60	4.38	*Imma*	6.15	8.84	6.15	335.2

Supplementary Table 13. Comparison of R_o, peak power densities of different single cells at different temperatures.

Electrolyte	Cathode	Thickness (μm)	R_o (Ω cm^2)	PPDs (mWcm^{-2})	Tem. (°C)	Ref.
BZCYYb	LSCF-BZCYYb	/	0.5	326	700	Ref. 2
BZCYYb	PBCCZ15-BZCYYb	17	0.3	449	650	Ref. 3
BZCYYb	SCF	38	0.29	551	650	Ref. 4
BZCYYb	PBC-BZCYYb	30	0.63	170	600	Ref. 5
BZCYYb	LSCF-BZCYYb	25	0.55	362	650	Ref. 6
BZCYYb	LSCF-BZCYYb	12	0.59	232	650	Ref. 7
BCZY7	LSN	15	0.28	330	650	Ref. 8
BZCYYb	BZFN	15	0.59	273	650	Ref. 9
BZCYYb-0.95	BCFZY	12	0.29	790	650	Ref. 10
BZCYYb	PBCCF5	20	0.27	946	650	This work
	PBCCF5	20	0.35	663	600	This work

Notes: BZCYYb=$BaZr_{0.1}Ce_{0.7}Y_{0.1}Yb_{0.1}O_{3-\delta}$; LSCF=$La_{0.6}Sr_{0.4}Co_{0.2}Fe_{0.8}O_{3-\delta}$; PBCCZ15=$PrBa_{0.9}Ca_{0.1}Co_{1.85}Zn_{0.15}O_{5+\delta}$; SCF=$SrCo_{0.8}Fe_{0.2}O_{3-\delta}$; BCZY7=$BaCe_{0.7}Zr_{0.1}Y_{0.2}O_{3-\delta}$; PBC=$PrBaCo_2O_{5+\delta}$; LSN=$Ln_{1.2}Sr_{0.8}NiO_4$; BZFN=$Ba_{0.9}Co_{0.7}Fe_{0.2}Nb_{0.1}O_{3-\delta}$; BCFZY= $BaCo_{0.4}Fe_{0.4}Zr_{0.1}Y_{0.1}O_{3-\delta}$.

Supplementary References

1. Dumschat J. Hochauflösende Röntgenabsorptionsspektroskopie an vierwertigen Seltene-Erdsystemen. *Department Physik*. Veröffentlichungen der Universität (2000).

2. Vafaeenezhad S, Sandhu NK, Hanifi AR, Etsell TH, Sarkar P. Development of proton conducting fuel cells using nickel metal support. *J. Power Sources* **435**, 226763 (2019).

3. Liu B*, et al.* Novel $PrBa_{0.9}Ca_{0.1}Co_{2\text{-}x}Zn_xO_{5+\delta}$ double-perovskite as an active cathode material for high-performance proton-conducting solid oxide fuel cells. *Int. J. Hydrog. Energ.* **45**, 31009-31016 (2020).

4. Lv X, Chen H, Zhou W, Li S-D, Shao Z. A CO_2-tolerant $SrCo_{0.8}Fe_{0.15}Zr_{0.05}O_{3-\delta}$ cathode for proton-conducting solid oxide fuel cells. *J. Mater. Chem. A* **8**, 11292-11301 (2020).

5. Liu B, Jia L, Chi B, Pu J, Li J. A novel $PrBaCo_2O_{5+\sigma}$-$BaZr_{0.1}Ce_{0.7}Y_{0.1}Yb_{0.1}O_3$ composite cathode for proton-conducting solid oxide fuel cells. *Compos. B. Eng.* **191**, 107936 (2020).

6. Hanifi AR, Sandhu NK, Etsell TH, Luo J-L, Sarkar P. Fabrication and characterization of a tubular ceramic fuel cell based on $BaZr_{0.1}Ce_{0.7}Y_{0.1}Yb_{0.1}O_{3-\delta}$ proton conducting electrolyte. *J. Power Sources* **341**, 264-269 (2017).

7. Chen X*, et al.* Fabrication and performance of anode-supported proton conducting solid oxide fuel cells based on $BaZr_{0.1}Ce_{0.7}Y_{0.1}Yb_{0.1}O_{3-\delta}$ electrolyte by multi-layer aqueous-based co-tape casting. *J. Power Sources* **506**, 229922 (2021).

8. Yang S, Wen Y, Zhang J, Lu Y, Ye X, Wen Z. Electrochemical performance and stability of cobalt-free $Ln_{1.2}Sr_{0.8}NiO_4$ (Ln=La and Pr) air electrodes for proton-conducting reversible solid oxide cells. *Electrochim. Acta* **267**, 269-277 (2018).

9. Wang S, Zhang L, Zhang L, Brinkman K, Chen F. Two-step sintering of ultrafine-grained barium cerate proton conducting ceramics. *Electrochim. Acta* **87**, 194-200 (2013).

10. He F*, et al.* Manipulating cation nonstoichiometry towards developing better electrolyte for self-humidified dual-ion solid oxide fuel cells. *J. Power Sources* **460**, 228105 (2020).
